# Associations between the C-reactive protein-triglyceride glucose index and the incidence and progression trajectory of cardiometabolic multimorbidity: a multi-state model study

**DOI:** 10.1186/s12933-026-03174-4

**Published:** 2026-04-05

**Authors:** Lei Yuan, Zhe Zhao, Hongxiang Peng, Wen Zong, Juanfang Zhu, Huilong Qu, Chun Liang, Jan Nilsson, Yihong Chen

**Affiliations:** 1 Faculty of Military Health Service, Naval Medical University, Shanghai, China; 2 Office of Academic Affairs, Naval Medical University, Shanghai, China; 3 Department of Cardiology, Second Affiliated Hospital of Naval Medical University, Shanghai, China; 4 Department of Clinical Sciences Malmö, Lund University, Malmö, 20502 Sweden; 5 Department of Experimental Medical Science, Lund University, Lund, 22184 Sweden

**Keywords:** C-reactive protein-triglyceride glucose index, Cardiometabolic multimorbidity, Type 2 diabetes, Stroke, Coronary heart diseases

## Abstract

**Background:**

The C-reactive protein-triglyceride-glucose index (CTI) has been proposed as a novel biomarker for insulin resistance and inflammation. However, its role in the progression trajectory of cardiometabolic multimorbidity (CMM) remains unclear. We aimed to investigate the involvement of the CTI in the CMM progression trajectory.

**Methods:**

This prospective study included 266,049 individuals from the UK Biobank, who were free of cardiometabolic diseases (CMD) at baseline. CMM was defined as the presence of two or more CMDs, including type 2 diabetes (T2D), coronary heart disease (CHD), and stroke. The CTI was calculated using the formula: 0.412 × ln (CPR) + ln (TG×FPG/2). Cox proportional hazards, Kaplan–Meier curves, restricted cubic spline (RCS) and multi-state models were employed to examine associations of CTI with the incidence and progression of CMM. Receiver operating characteristic (ROC) curve, C-index analysis, net reclassification index (NRI) together with integrated discrimination improvement index (IDI) were carried out to examinate the predictive performance of CTI. The robustness of results was further evaluated via stratified and sensitivity analyses.

**Results:**

CTI was positively and significantly associated with CMM development. Compared with the low-CTI group, the high-CTI group exhibited an increased risks of T2D (HR: 3.60, 95% CI 3.39–3.83), stroke (HR: 1.11, 95% CI 1.03–1.19), CHD (HR: 1.52, 95% CI 1.46–1.58), first cardiometabolic disease (FCMD, HR: 1.86, 95% CI 1.81–1.92), CMM (HR: 2.50, 95% CI 2.23–2.80), and death (HR: 1.25, 95% CI 1.20–1.29). Among CMM and its component diseases, CTI showed the greater predictive capacity for T2D and CMM risk. Additionally, CTI exhibited incremental predictive value over TyG and CRP for incident CHD, FCMD and CMM with the highest C-index and NRI values. Stratified analyses indicated the consistent association of CTI with all outcomes except for stroke across age, gender and BMI. Specifically, stronger associations were observed in younger, female and lower BMI individuals. In state transition analysis, the high-CTI group showed elevated risks for transitions from baseline to FCMD (HR: 1.86, 95% CI 1.80–1.91), baseline to death (HR: 1.18, 95% CI 1.12–1.23), and FCMD to CMM (HR: 1.39, 95% CI 1.24–1.56). In disease-specific transitions, a higher CTI was linked to increased risks of transitions from baseline to T2D (HR: 3.68, 95% CI 3.45–3.93), baseline to CHD (HR: 1.49, 95% CI 1.43–1.56), baseline to death (HR: 1.18, 95% CI 1.12–1.23), stroke to CMM (HR: 1.43, 95% CI 1.09–1.86), and CHD to CMM (HR: 1.52, 95% CI 1.29–1.79). Similar findings were observed when the CTI was treated as a continuous variable.

**Conclusion:**

Our data revealed that CTI was positively correlated with the incidence and progression trajectory of CMM. CTI could serve as a simple and scalable tool for risk stratification in CMM, highlighting its potential utility in screening population with cardiometabolic-inflammatory burden.

**Graphical abstract:**

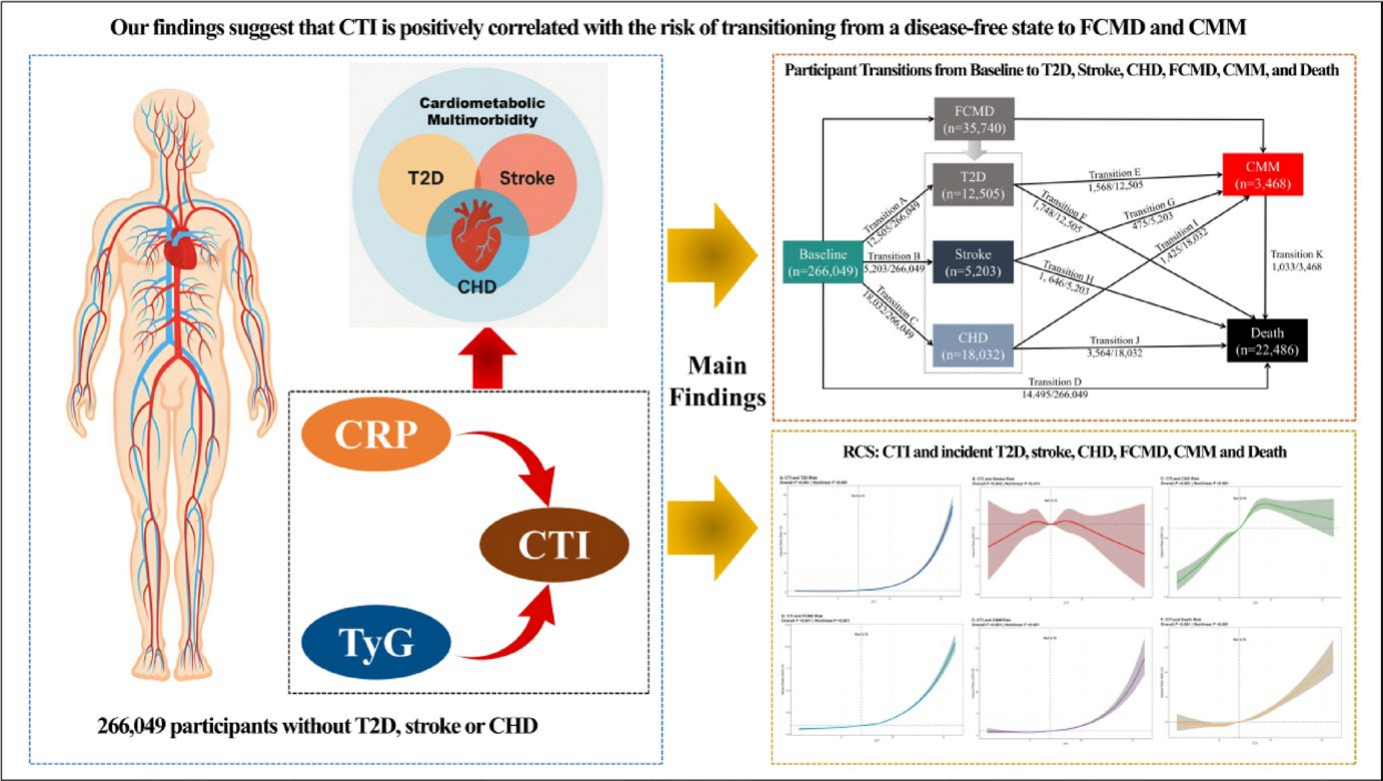

**Supplementary Information:**

The online version contains supplementary material available at 10.1186/s12933-026-03174-4.

## Introduction

Cardiometabolic multimorbidity (CMM) refers to the occurrence of two or more diseases, such as type 2 diabetes (T2D), stroke, and coronary heart disease (CHD), and is considered a serious comorbidity [1–3]. The risk of CMM increases, particularly among individuals aged 65 years and older, leading to significant impairments in quality of life and life expectancy. It is also a major contributor to global mortality [4–7]. With an aging global population, projections suggest that, by 2100, there will be approximately 2.37 billion adults aged 65 years and older worldwide [8]. This demographic trend suggests that the burden of CMM will not only grow substantially in terms of the aging population affected but will also intensify its economic impact by escalating healthcare expenditures and productivity losses [2, 9, 10].

Studies have indicated that insulin resistance (IR), which reflects the diminished physiological effectiveness of insulin, is a common pathological mechanism underlying various metabolic disorders [11–13]. It is closely related to atherosclerosis onset and progression of and serves as an important indicator for the progression of CMM diseases [14]. Previous studies have demonstrated the significance of insulin and related biomarkers in predicting CMM development [11, 13, 15]. Due to increased inflammation, oxidative stress, and endothelial dysfunction, IR may trigger arterial stiffness and cardiovascular diseases (CVDs) [16]. The triglyceride-glucose (TyG) index effectively measures insulin sensitivity and is widely applied in clinical practice [17, 18]. An increasing body of evidence has shown that the TyG index is significantly associated with atherosclerosis, stroke, and CVD outcomes [11, 12, 19]. Furthermore, studies have found that inflammation promotes arterial stiffness, damages endothelial integrity, and increases thrombosis, thereby significantly increasing the risk of CMM [20–23]. C-reactive protein (CRP) has been shown to be significantly associated with stroke risk and is a proven practical biomarker for evaluating stroke events [21, 24, 25]. However, existing studies on the C-reactive protein-triglyceride glucose index (CTI) have primarily focused on single cardiometabolic diseases, demonstrating superior assessment performance, compared with either CRP or the TyG index alone [24, 26]. This is because relying solely on either the TyG index or CRP captures only one aspect of potential pathological changes. In contrast, the clinical utility of the CTI is enhanced through three synergistic mechanisms, providing a more comprehensive evaluation, compared with isolated biomarkers, such as the TyG index or CRP. Despite these advantages, the role of the CTI in CMM and its progression remain underexplored, and our understanding of how the CTI influences transitions within the context of CMM remains limited.

Therefore, in this study, we utilized long-term follow-up data from the UK Biobank (UKB) to analyze the role of the CTI in CMM progression. By adopting the CTI algorithm from previous studies, this study sought to provide further evidence to support the real-world applicability of the CTI in CMM risk stratification and disease progression.

## Methods

### Study population

**Fig. 1:**
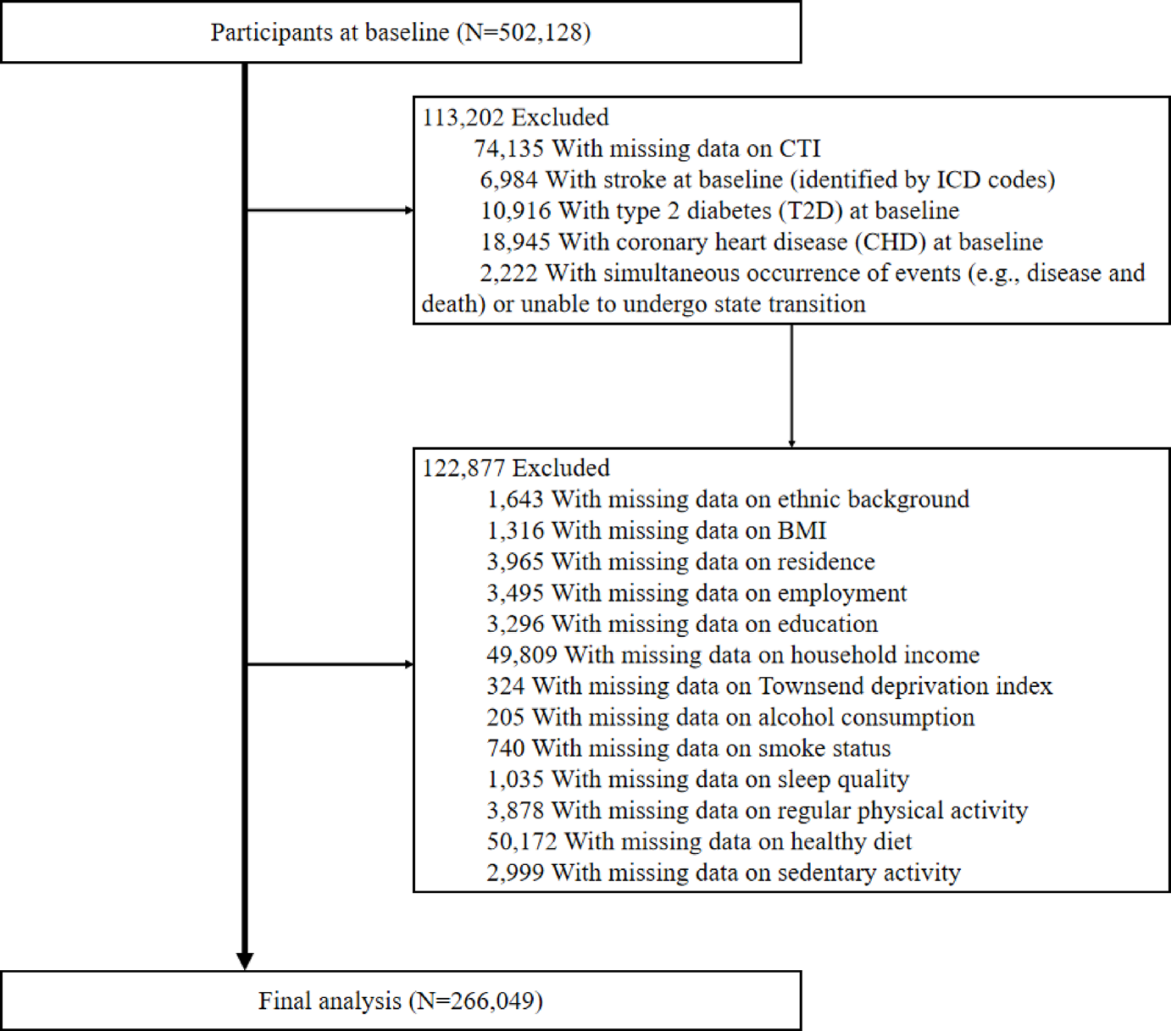
Flowchart of participants selection. ICD: International Classification of Diseases; T2D: type 2 diabetes; CHD: coronary heart disease; BMI: body mass index

In this study, we utilized data from the UKB, a cohort study involving data tracking and follow-up from the baseline period between 2006 and 2010 [27]. The UKB database includes data on a wide range of variables, such as participants’ demographic characteristics, lifestyle habits, socioeconomic status, environmental factors, and healthcare access, along with data regarding biological samples collected, such as blood, saliva, and urine. This study was approved by the Ethics Committee of the Northwest Multi-Center Research, and all participants provided written informed consent. The research was conducted under UKB application number 99709. The inclusion criteria for the study participants are outlined in Fig. 1, and data from a total of 266,049 participants were included in the analysis.

## Assessment of the CTI

Inflammation was quantified using CRP, and IR was assessed using the TyG index. These parameters were combined to form the CRP-TyG index, which incorporated the CRP level (mg/L) and TyG index (calculated from triglyceride [TG, mg/dL] and fasting plasma glucose [FPG, mg/dL]) levels. The CTI was calculated using the following formula [28]:

$$\:CTI=0.412\times\:\mathrm{ln}\:\left(\mathrm{C}\mathrm{P}\mathrm{R}\right)\:+\mathrm{ln}\:(\mathrm{T}\mathrm{G}\times\:\mathrm{F}\mathrm{P}\mathrm{G}/2)$$

We divided the participants into the following groups according to the CTI tertile values: a low-CTI group (CTI < 8.4875), a medium-CTI group (8.4875–9.1968), and a high-CTI group (> 9.1968).

## Outcome ascertainment

Baseline diagnoses of T2D, stroke, and CHD were based on clinical records and self-reported information, including T2D (International Classification of Diseases, 10th Revision [ICD-10] code: E11), stroke (ICD-10 codes: I60-I64 and I69), and CHD (ICD-10 codes: I20-I25). The incidence of T2D, stroke, and CHD during follow-up was determined using the UKB algorithm [29, 30]. This algorithm integrates data from self-reports, hospital admissions, death registries, and primary care records. The follow-up period was defined as the time from recruitment to the first diagnosis of T2D, stroke, or CHD; loss to follow-up; death; or data censoring, whichever occurred first.

First cardiometabolic disease (FCMD) was defined as the initial diagnosis of any cardiometabolic disease, whereas CMM referred to the coexistence of at least two of these diseases. FCMD onset was recorded as the time of the first diagnosis of cardiometabolic disease, and death was recorded based on the date of death. Cardiometabolic diseases included T2D, stroke, and CHD. CMM onset was defined as the occurrence of the second cardiometabolic disease (CMD). Participants who experienced simultaneous occurrence of two diseases during follow-up or had FCMD and death or CMM and death occurring simultaneously were excluded from the analysis because they did not meet the criteria for the state-transition model [31, 32].

## Covariate ascertainment

Covariates were selected basing on previous cardiovascular studies grounded in causal inference frameworks using the UK Biobank and included demographic characteristics, socioeconomic status, and lifestyle factors that may influence the development of CVD and CMM [1, 6, 11, 26]. Covariates used in this study were age, sex, ethnicity, body mass index (BMI), place of residence, employment status, education level, household income, Townsend deprivation index, alcohol consumption, smoking status, sleep quality, participation in regular physical activity, adherence to a healthy diet, and sedentary behaviour. Further details are provided in Supplementary Table S1–S3. Additional data and resources can be accessed on the UKB website (www.ukbiobank.ac.uk).

### Statistical analysis

Participants’ baseline characteristics were categorised based on the CTI tertiles. For continuously skewed variables, the median and interquartile range were reported, whereas categorical variables were presented as frequencies and percentages (%). Group differences were analysed using the Kruskal–Wallis H test for continuous variables and the chi-square test for categorical variables.Cox proportional hazards regression analysis was used to calculate the hazard ratios (HR) and 95% confidence intervals (CI) for the risk of T2D, stroke, CHD, FCMD, CMM, and death based on the CTI (both as a continuous variable and grouped into tertiles). The crude model refers to the unadjusted model, whereas the adjusted model 1 was controlled for age, sex, ethnicity, and BMI; the adjusted model 2 was further controlled for the place of residence, employment status, education level, household income, and Townsend deprivation index; and the adjusted model 3 was additionally controlled for alcohol consumption, smoking status, sleep quality, regular physical activity, adherence to a healthy diet, and healthy sedentary behavior. Additionally, restricted cubic splines with five knots were used to visualize the dose-response relationship between the CTI and outcomes. C-index was carried out to compare the predictive value of CTI for risk of all outcomes.

Net reclassification index (NRI) and integrated discrimination improvement index (IDI) were performed to quantify the incremental predictive value of CTI, TyG index and CRP compared to the fully adjusted model. Stratified analyses across age, sex and BMI were used to test the consistency of correlation and control confounding.

**Fig. 2:**
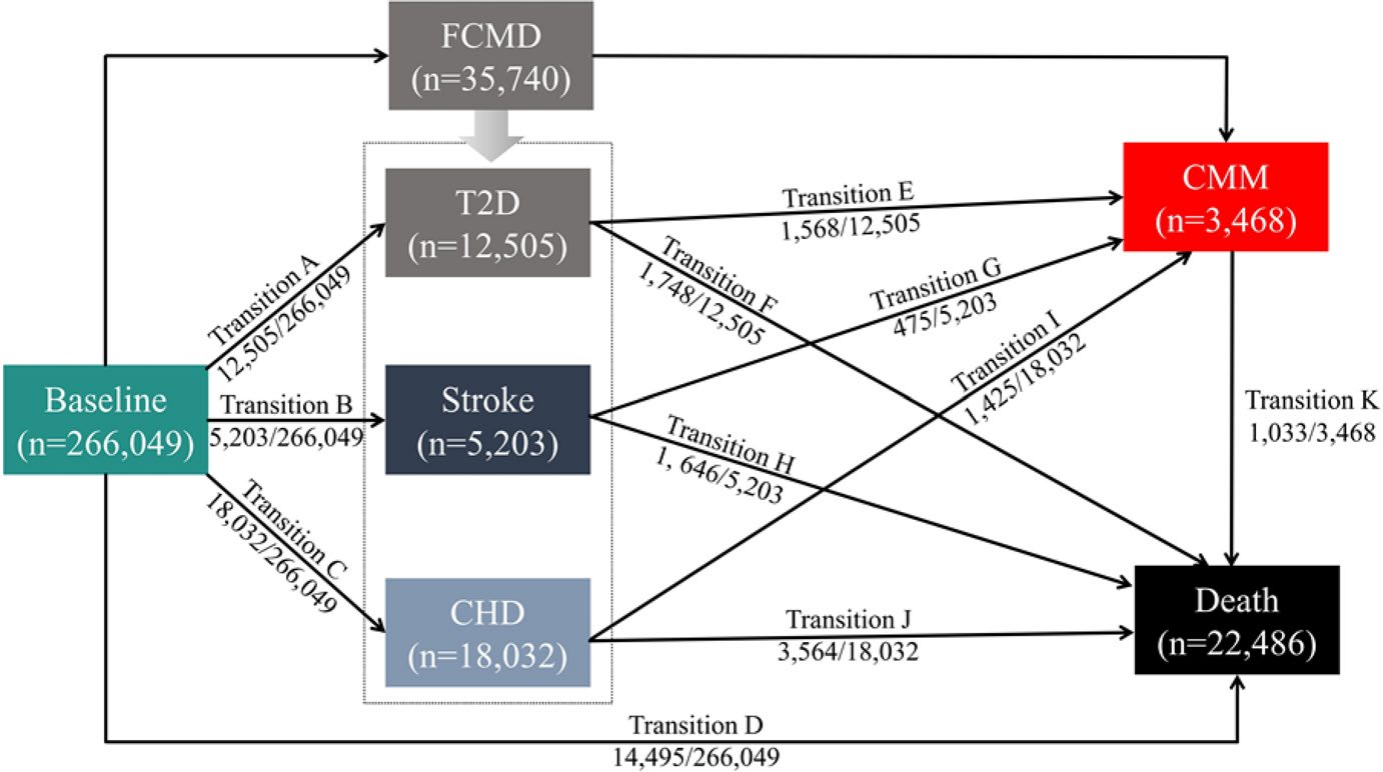
Participant Transitions from Baseline to T2D, Stroke, CHD, FCMD, CMM, and Death. T2D: type 2 diabetes; CHD: coronary heart disease; FCMD: first cardiometabolic disease; CMM: cardiometabolic multimorbidity

The multi-state model, an extension of the competing risks model, was used to comprehensively assess the impact of risk factors on progression across different disease stages. This method has been previously described [33, 34]. In this study, the multi-state model was used to analyze the role of the CTI in the progression of FCMD, from no FCMD to CMM. As shown in Fig. 2, two pathways were constructed based on the progression of CMM comorbidity: Pathway 1 consists of five transition stages: (A) baseline to FCMD, (B) baseline to death, (C) FCMD to CMM, (D) FCMD to death, and (E) CMM to death. Pathway 2 includes 11 transition stages: (A) baseline to T2D, (B) baseline to stroke, (C) baseline to CHD, (D) baseline to death, (E) T2D to CMM, (F) T2D to death, (G) stroke to CMM, (H) stroke to death, (I) CHD to CMM, (J) CHD to death, and (K) CMM to death.

Three sensitivity analyses were performed to assess the robustness of the results: (1) excluding participants who developed diseases within the first 2 years of the study, then reanalysing the multi-state model and CMM-related disease risk; (2) reanalysing the CTI and CMM-related disease risk with death as a competing risk; and (3) reanalysing CMM-related disease incidence after imputing missing values using chained equations (*n* = 5). All analyses were conducted using R (version 4.4.1) and Stata MP17.0, with the ‘mstate’ package employed for multi-state modelling. Statistical tests were two-sided, with significance set at *P* < 0.05.

## Results

### Baseline characteristics of participants

The baseline characteristics of the study participants are summarized in Table 1. A total of 266,049 individuals were included in the study, with a mean age of 55.85 years (standard deviation [SD] ± 8.05), and women accounted for 54.78%. The average CTI was 8.79 (SD ± 0.78). Participants in high-CTI group tended to be older, and were more likely to be male, retired, have a lower household income, and have a higher BMI. During a median follow-up of 14.78 years, 3,468 participants developed CMM, and 22,486 participants died.

**Table 1:** Baseline information of participants stratified based on the CTI

Variables	Low-CTI group (*N* = 95,354)	Medium-CTI group (*N* = 89,219)	High-CTI group (*N* = 81,476)	*P* value
Age, Median (P_25_, P_75_) ^a^	54 (47,61)	58 (50,63)	58 (51,63)	< 0.001
Sex				< 0.001
Male	34,652(36.34)	42,731(47.89)	42,920(52.68)	
Female	60,702(63.66)	46,488(52.11)	38,556(47.32)	
Ethnicity				< 0.001
White	91,186(95.63)	85,795(96.16)	78,532(96.39)	
Mixed	623(0.65)	477(0.53)	380(0.47)	
Asian	920(0.96)	1201(1.35)	1336(1.64)	
Black	1689(1.77)	967(1.08)	556(0.68)	
Chinese	323(0.34)	201(0.23)	137(0.17)	
Other	613(0.64)	578(0.65)	535(0.66)	
Place of residence				< 0.001
Urban	81,238(85.20)	75,966(85.15)	70,060(85.99)	
Rural	14,116(14.80)	13,253(14.85)	11,416(14.01)	
Employment status				< 0.001
Employed	69,584(72.97)	56,730(63.59)	48,657(59.72)	
Retired	20,505(21.50)	27,817(31.18)	27,083(33.24)	
Unemployed	5265(5.52)	4672(5.24)	5736(7.04)	
Education level				< 0.001
College or above	66,065(69.28)	56,827(63.69)	48,459(59.48)	
High school or equivalent	5830(6.11)	4988(5.59)	4531(5.56)	
Less than high school	23,459(24.60)	27,404(30.72)	28,486(34.96)	
Household income				< 0.001
< 18,000£	14,335(15.03)	18,287(20.50)	20,405(25.04)	
18,000–30,999£	21,945(23.01)	23,152(25.95)	22,030(27.04)	
31,000–51,999£	27,092(28.41)	24,282(27.22)	21,014(25.79)	
52,000–99,999£	24,511(25.71)	18,818(21.09)	14,814(18.18)	
≥ 100,000£	7471(7.84)	4680(5.25)	3213(3.94)	
BMI (kg/m^2^)				< 0.001
< 18.5	54,910(57.59)	26,057(29.21)	11,128(13.66)	
18.5–24.9	1125(1.18)	208(0.23)	59(0.07)	
25.0-29.9	33,268(34.89)	44,354(49.71)	36,842(45.22)	
≥ 30.0	6051(6.35)	18,600(20.85)	33,447(41.05)	
Townsend deprivation index				< 0.001
Good	34,805(36.50)	32,170(36.06)	27,196(33.38)	
General	32,348(33.92)	30,741(34.46)	27,266(33.47)	
Poor	28,201(29.58)	26,308(29.49)	27,014(33.16)	
Alcohol consumption				< 0.001
Never	2770(2.90)	2984(3.34)	3271(4.01)	
Ever	2586(2.71)	2635(2.95)	2964(3.64)	
Current	89,998(94.38)	83,600(93.70)	75,241(92.35)	
Smoking status				< 0.001
Never	58,249(61.09)	49,865(55.89)	41,016(50.34)	
Ever	30,181(31.65)	31,197(34.97)	30,420(37.34)	
Current	6924(7.26)	8157(9.14)	10,040(12.32)	
Sleep quality				< 0.001
Bad	26,320(27.60)	26,907(30.16)	27,361(33.58)	
Good	69,034(72.40)	62,312(69.84)	54,115(66.42)	
Regular physical activity				< 0.001
No	14,806(15.53)	17,142(19.21)	19,902(24.43)	
Yes	80,548(84.47)	72,077(80.79)	61,574(75.57)	
Adherence to a healthy diet				< 0.001
No	65,352(68.54)	65,209(73.09)	63,089(77.43)	
Yes	30,002(31.46)	24,010(26.91)	18,387(22.57)	
Healthy sedentary activity				< 0.001
No	13,201(13.84)	17,482(19.59)	21,094(25.89)	
Yes	82,153(86.16)	71,737(80.41)	60,382(74.11)	

^a^denotes variables (age) analysed with the Kruskal–Wallis test due to non-normal distribution; other categorical variables were tested using the chi-square test

CTI, C-reactive protein-triglyceride-glucose index; BMI, body mass index

## The CTI and risk of diseases associated with CMM

**Fig. 3:**
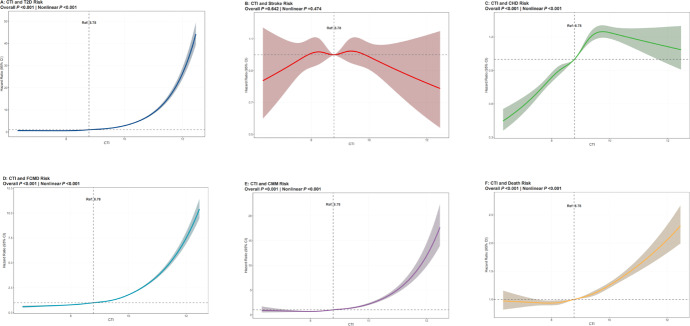
Restricted cubic spline analyses of the association between CTI and incident T2D, stroke, CHD, CMM and Death. *Note*: All models were adjusted for age, sex, ethnicity, BMI, place of residence, employment status, education level, household income, Townsend deprivation index, alcohol consumption, smoking status, sleep quality, regular physical activity, adherence to a healthy diet and healthy sedentary activity. CTI: C-reactive protein-triglyceride-glucose index; T2D: type 2 diabetes; CHD: coronary heart disease; CMM: cardiometabolic multimorbidity; BMI: body mass index

Table 2 presents the associations of the CTI with T2D, stroke, CHD, FCMD, CMM, and death. The Kaplan–Meier curves illustrating these associations are displayed in Supplementary Figure S1. In the fully adjusted model, compared with the low-CTI group, the medium- and high-CTI groups showed a significantly increased risk of T2D, stroke, CHD, FCMD, CMM, and death. Specifically, compared with the low-CTI group, the HR and 95% CI for the high-CTI group for T2D, stroke, CHD, FCMD, CMM, and death were 3.60 (3.39, 3.83), 1.11 (1.03, 1.19), 1.52 (1.46, 1.58), 1.86 (1.81, 1.92), 2.50 (2.23, 2.80), and 1.25 (1.20, 1.29), respectively. Furthermore, when the CTI was treated as a continuous variable, the results showed that, for every 1-point increase in the CTI, the risk of T2D, stroke, CHD, FCMD, CMM, and death increased by 142%, 8%, 26%, 56%, 90%, and 16%, respectively. The dose-response relationships between CTI and T2D, stroke, CHD, FCMD, CMM, and death were further explored using Restricted Cubic Spline (RCS) analysis (Fig. 3). The results showed a significant positive association between CTI and disease outcomes in all groups (Overall P-value < 0.001; Nonlinear P-value < 0.001), characterized by gradual risk increases at lower CTI levels followed by sharp increases once CTI exceeded a certain threshold, a pattern not observed for stroke.

**Table 2:** Association between the CTI groups and T2D, stroke, CHD, FCMD, CMM, and death

Category	Crude model		Adjusted model 1		Adjusted model 2		Adjusted model 3	
	HR (95%CI)	*P* value	HR (95%CI)	*P* value	HR (95%CI)	*P* value	HR (95%CI)	*P* value
CTI as a categorical variable (three groups; ref.: low)								
T2D								
Medium	2.81 (2.64,2.99)	< 0.001	1.76 (1.65,1.88)	< 0.001	1.72 (1.61,1.84)	< 0.001	1.69 (1.59,1.81)	< 0.001
High	8.37 (7.91,8.86)	< 0.001	3.97 (3.74,4.22)	< 0.001	3.76 (3.54,4.00)	< 0.001	3.60 (3.39,3.83)	< 0.001
Stroke								
Medium	1.39 (1.30,1.48)	< 0.001	1.08 (1.01,1.15)	0.021	1.06 (0.99,1.13)	0.074	1.04 (0.98,1.11)	0.224
High	1.64 (1.54,1.75)	< 0.001	1.21 (1.13,1.29)	< 0.001	1.16 (1.08,1.24)	< 0.001	1.11 (1.03,1.19)	0.004
CHD								
Medium	1.73 (1.67,1.80)	< 0.001	1.29 (1.24,1.34)	< 0.001	1.27 (1.22,1.32)	< 0.001	1.25 (1.20,1.30)	< 0.001
High	2.45 (2.36,2.54)	< 0.001	1.64 (1.57,1.70)	< 0.001	1.58 (1.52,1.64)	< 0.001	1.52 (1.46,1.58)	< 0.001
FCMD								
Medium	1.84 (1.78,1.89)	< 0.001	1.32 (1.28,1.36)	< 0.001	1.29 (1.25,1.33)	< 0.001	1.27 (1.23,1.31)	< 0.001
High	3.31 (3.22,3.40)	< 0.001	2.02 (1.96,2.09)	< 0.001	1.94 (1.88,2.00)	< 0.001	1.86 (1.81,1.92)	< 0.001
CMM								
Medium	2.49 (2.22,2.79)	< 0.001	1.52 (1.35,1.71)	< 0.001	1.47 (1.31,1.66)	< 0.001	1.44 (1.28,1.61)	< 0.001
High	5.99 (5.40,6.65)	< 0.001	2.87 (2.57,3.22)	< 0.001	2.66 (2.38,2.98)	< 0.001	2.50 (2.23,2.80)	< 0.001
Death								
Medium	1.47 (1.42,1.52)	< 0.001	1.15 (1.11,1.19)	< 0.001	1.11 (1.07,1.15)	< 0.001	1.08 (1.04,1.12)	< 0.001
High	1.97 (1.90,2.03)	< 0.001	1.44 (1.39,1.49)	< 0.001	1.35 (1.30,1.40)	< 0.001	1.25 (1.20,1.29)	< 0.001
CTI as continues								
T2D	3.31 (3.24,3.38)	< 0.001	2.56 (2.50,2.63)	< 0.001	2.48 (2.42,2.54)	< 0.001	2.42 (2.36,2.48)	< 0.001
Stroke	1.32 (1.28,1.37)	< 0.001	1.14 (1.10,1.18)	< 0.001	1.11 (1.07,1.15)	< 0.001	1.08 (1.04,1.12)	< 0.001
CHD	1.59 (1.57,1.62)	< 0.001	1.33 (1.30,1.35)	< 0.001	1.29 (1.27,1.32)	< 0.001	1.26 (1.24,1.29)	< 0.001
FCMD	2.01 (1.98,2.04)	< 0.001	1.64 (1.62,1.67)	< 0.001	1.60 (1.58,1.62)	< 0.001	1.56 (1.54,1.59)	< 0.001
CMM	2.68 (2.57,2.79)	< 0.001	2.08 (1.98,2.18)	< 0.001	1.98 (1.89,2.08)	< 0.001	1.90 (1.81,2.00)	< 0.001
Death	1.46 (1.44,1.49)	< 0.001	1.27 (1.25,1.30)	< 0.001	1.22 (1.20,1.24)	< 0.001	1.16 (1.14,1.18)	< 0.001

T2D, stroke, and CHD refer to the disease incidence over the entire follow-up period, rather than the first occurrence of disease assessed in the FCMD, which differs from the calculation method shown in Fig. 2. The crude model refers to the unadjusted model, whereas the adjusted model 1 was controlled for age, sex, ethnicity, and BMI; the adjusted model 2 was further controlled for the place of residence, employment status, education level, household income, and Townsend deprivation index; and the adjusted model 3 was additionally controlled for alcohol consumption, smoking status, sleep quality, regular physical activity, adherence to a healthy diet, and healthy sedentary behavior

CTI, C-reactive protein-triglyceride-glucose index; T2D, type 2 diabetes; CHD, coronary heart disease; FCMD, first cardiometabolic disease; CMM, cardiometabolic multimorbidity; BMI, body mass index

### The predictive value of CTI for diseases associated with CMM

To determine the predictive value of the CTI, the Receiver operating characteristic (ROC) curve analysis was carried out. In ROC curve analysis (Supplementary Figure S2), CTI displayed the highest predictive accuracy for predicting T2D risk with area under the curve (AUC) value of 0.751, followed by 0.710 for CMM, 0.657 for FCMD, 0.610 for CHD and 0.589 for death. The optimal CTI cutoff value and corresponding sensitivity and specificity were shown in Supplementary Figure S2. To further address if CTI can offer incremental predictive value over CRP and TyG, the C-index, net reclassification index (NRI) and integrated discrimination improvement index (IDI) analyses were performed. In summary, CTI showed slightly superior predictive performance than CRP and TyG alone in most CMM-related diseases. In C-index analysis (Table 3), the CTI had the greatest predictive value for the risk of CHD, FCMD, and CMM with C-index values of 0.715, 0.729, and 0.816 respectively, in comparison with the TyG index (0.713, 0.726, and 0.813) and CRP (0.712, 0.718, and 0.801). For the incidence of T2D, CTI and the TyG index displayed the comparable predictive performance (0.808 and 0.809), but both of them significantly higher than for CRP (0.778). For the risk of death, CTI was similar to CRP (0.743 and 0.744), slightly outperforming the TyG index (0.741). No significant differences were observed among three indicators for stroke. Moreover, NRI and IDI analyses (Supplementary Table S4) confirmed that incorporating CTI into full adjusted model results in the greatest improvement in predicting CHD, FCMD, and CMM with INR values of 0.093 (95% CI 0.081–0.105), 0.158 (95% CI 0.149–0.168) and 0.216 (95% CI 0.167–0.262), followed by the TyG index with 0.072 (95% CI 0.058–0.084), 0.143 (95% CI 0.132–0.153) and 0.200 (95% CI 0.144–0.246), and both were clearly superior to CRP with 0.056 (95% CI 0.044,0.067), 0.076 (95% CI 0.066,0.085) and 0.091 (95% CI 0.052,0.144). The IDI values were small, but CTI remained the highest (0.007, 95% CI 0.007–0.008), outperforming the TyG index (0.006) and CRP (0.001). For T2D, stroke, and death, the INR and IDI values of the CTI were comparable to those of the best-performing indicators (the TyG index or CRP). Our data, in accordance with results from recent studies [35–37], suggesting that CTI may provide the incremental predictive value beyond its individual components (TyG and CRP) in predicting incidence of CMM and related diseases.

**Table 3:** Comparison of C-index values among different indices (CTI, TyG, and CRP)

Outcomes	CTI		TyG		CRP	
	95% CI	*P* value	95% CI	*P* value	95% CI	*P* value
T2D	0.808 (0.805,0.812)	Ref.	0.809 (0.806,0.812)	0.277	0.778 (0.775,0.782)	< 0.001
Stroke	0.706 (0.700,0.713)	Ref.	0.706 (0.700,0.712)	0.108	0.707 (0.701,0.713)	0.050
CHD	0.715 (0.712,0.718)	Ref.	0.713 (0.710,0.716)	< 0.001	0.712 (0.708,0.715)	< 0.001
FCMD	0.729 (0.726,0.731)	Ref.	0.726 (0.724,0.729)	< 0.001	0.718 (0.715,0.720)	< 0.001
CMM	0.816 (0.809,0.822)	Ref.	0.813 (0.806,0.819)	< 0.001	0.801 (0.795,0.808)	< 0.001
Death	0.743 (0.740,0.746)	Ref.	0.741 (0.738,0.745)	< 0.001	0.744 (0.741,0.747)	< 0.001

CTI, TyG, and CRP as continuous variables

### Stratification of CTI in relation to diseases associated with CMM

To further evaluate the robustness of association between CTI and CMM related diseases and to construct an implementation framework for clinical practice, we performed stratified analyses according to age (< 60 or ≥ 60 years old), sex and BMI (< 30 or ≥ 30 kg/m^2^), in crude model and fully adjusted model (Supplementary Table S5–S7). Overall, the positive association between CTI level and risk of all disease outcomes, except for stroke, was directionally consistent across all predefined subgroups after controlling for variable. As shown in Supplementary Table S5, in age-stratified analysis, younger individuals showed steeper CTI level-associated risk gradient for incidence of T2D, CHD, FCMD, CMM, and death compared to elderly individuals (all *P* < 0.001). For stroke, CTI had a mild dose–response relationship in younger individuals, but no association in elderly individuals. In sex-stratified and BMI-stratified analysis (Supplementary Table S6–S7), a stronger association between CTI and risk of T2D, CHD, FCMD, CMM, and death was observed in female and lower BMI individuals (all *P* < 0.001). Notably, CTI had the highest association with T2D risk (Female: HR 5.13, 95% CI 4.65–5.66; lower BMI: HR 4.60, 95% CI 4.28–4.94), followed by CMM risk (Female: HR 3.58, 95% CI 2.94–4.36; lower BMI: HR 3.11, 95% CI 2.74–3.54) in those individuals. A statistically significant association between CTI and stroke only observed in female and lower BMI individuals with the highest CTI level, but the estimated risk increases were modest (Female: HR 1.14, 95% CI 1.02–1.47; lower BMI: HR 1.14, 95% CI 1.06–1.23).These findings indicated that the relationship between CTI and all outcomes except for stroke, is broadly stable across these strata. Importantly, CTI showed the stronger disease prediction in younger and female individuals with lower BMI.

### State transition analysis of the CTI and CMM-related diseases

Table 4 presents the associations of the CTI with the transitions from baseline to FCMD, CMM, and death. In the fully adjusted model, compared with the low-CTI group, the medium- and high-CTI groups showed significantly increased risks for transitions from baseline to FCMD, baseline to death, and FCMD to CMM. Specifically, the high-CTI group demonstrated increased risks of 86% (HR: 1.86, 95% CI 1.80–1.91), 18% (HR: 1.18, 95% CI 1.12–1.23), and 39% (HR: 1.39, 95% CI 1.24–1.56), respectively. When the CTI was treated as a continuous variable, for each additional point of the CTI, the risks for transitions from baseline to FCMD, baseline to death, and FCMD to CMM increased by 56% (HR: 1.56, 95% CI 1.53–1.58), 11% (HR: 1.11, 95% CI 1.09–1.14), and 22% (HR: 1.22, 95% CI 1.17–1.28), respectively.

**Table 4:** Associations of the CTI with the transitions from baseline to FCMD, CMM, and Death

Groups	Baseline → FCMD	Baseline → Death	FCMD → CMM	FCMD → Death	CMM → Death
CTI as a categorical variable (three levels; ref.: low)					
Medium	1.27 (1.23,1.31) ***	1.06 (1.02,1.11) **	1.15 (1.02,1.29) *	0.91 (0.84,0.99) *	1.00 (0.81,1.24)
High	1.86 (1.80,1.91) ***	1.18 (1.12,1.23) ***	1.39 (1.24,1.56) ***	0.91 (0.84,0.99) *	1.02 (0.84,1.25)
CTI as a continuous variable					
CTI	1.56 (1.53,1.58) ***	1.11 (1.09,1.14) ***	1.22 (1.17,1.28) ***	0.98 (0.94,1.01)	1.00 (0.92,1.09)

*** *P* < 0.001, **P < 0.01, * *P* < 0.05

All models were adjusted for age, sex, ethnicity, BMI, place of residence, employment status, education level, household income, Townsend deprivation index, alcohol consumption, smoking status, sleep quality, regular physical activity, adherence to a healthy diet, and healthy sedentary activity

CTI, C-reactive protein-triglyceride-glucose index; FCMD, first cardiometabolic disease; CMM, cardiometabolic multimorbidity

Table 5 presents the transition risks for specific diseases related to FCMD. Compared with the low-CTI group, the high-CTI group demonstrated significantly increased risks for the transitions from baseline to T2D, baseline to CHD, baseline to death, stroke to CMM, and CHD to CMM, with increases of 268% (HR: 3.68, 95% CI 3.45–3.93), 49% (HR: 1.49, 95% CI 1.43–1.56), 18% (HR: 1.18, 95% CI 1.12–1.23), 43% (HR: 1.43, 95% CI 1.09–1.86), and 52% (HR: 1.52, 95% CI 1.29–1.79), respectively. A similar pattern was observed when the CTI was treated as a continuous variable. The C-index values of the CTI across different state transitions are presented in Supplementary Tables S8 and S9, demonstrating its favourable predictive performance for each disease transition and its potential as a novel indicator for predicting the incidence, prognosis, and progression of CMM among healthy populations or patients with CVD in clinical practice.

**Table 5:** Associations of the CTI with transitions from baseline to T2D, stroke, CHD, CMM, and Death

Transition path	CTI as a categorical variable (three levels; ref.: low)		CTI as a continuous variable
	Medium	High	
Baseline → T2D	1.70 (1.59,1.82) ***	3.68 (3.45,3.93) ***	2.47 (2.40,2.53) ***
Baseline → stroke	1.02 (0.95,1.10)	1.06 (0.98,1.14)	1.04 (1.00,1.08)
Baseline → CHD	1.24 (1.19,1.29) ***	1.49 (1.43,1.56) ***	1.25 (1.22,1.28) ***
Baseline → Death	1.06 (1.02,1.11) **	1.18 (1.12,1.23) ***	1.11 (1.09,1.14) ***
T2D → CMM	1.09 (0.88,1.33)	1.11 (0.92,1.35)	1.06 (1.00,1.14)
T2D → Death	0.89 (0.74,1.07)	0.86 (0.72,1.02)	0.95 (0.89,1.01)
Stroke → CMM	1.21 (0.93,1.57)	1.43 (1.09,1.86) **	1.22 (1.07,1.40) **
Stroke → Death	0.86 (0.74,0.99) *	0.86 (0.74,1.00) *	0.93 (0.86,1.01)
CHD → CMM	1.12 (0.94,1.33)	1.52 (1.29,1.79) ***	1.41 (1.30,1.52) ***
CHD → Death	1.01 (0.90,1.13)	1.01 (0.90,1.14)	1.01 (0.95,1.08)
CMM → Death	1.00 (0.81,1.24)	1.02 (0.84,1.25)	1.00 (0.92,1.09)

*** *P* < 0.001, **P < 0.01, * *P* < 0.05

All models were adjusted for age, sex, ethnicity, BMI, place of residence, employment status, education level, household income, Townsend deprivation index, alcohol consumption, smoking status, sleep quality, regular physical activity, adherence to a healthy diet and healthy sedentary activity

CTI: C-reactive protein-triglyceride-glucose index; T2D: type 2 diabetes; CMM: cardiometabolic multimorbidity; BMI: body mass index

### Sensitivity analysis

The sensitivity analysis showed similar results when excluding participants who developed diseases within the first 2 years of the study, considering death as a competing risk, or using chained imputation (*n* = 5) for missing data. These findings support the robustness of our conclusions (Supplementary Table S10–S14).

## Discussion

This large-scale prospective study revealed a significant association between the CTI and increased risk of CMM progression. CTI was positively associated with component diseases of CMM including T2D, stroke, CHD and FCMD, together with mortality. We found a clear does-relationship between CTI level and all outcomes except for stroke. Crucially, CTI showed superior predictive performance for the initial CHD, FCMD, and CMM compared with its individual components TyG and CRP alone, suggesting the importance of assessing metabolic dysregulation and systemic inflammation integrally in long-tern CVD management. In sex-specific, age- and BMI stratified subgroups, the positive correlations between CTI and risk of T2D, CHD, FCMD, CMM and death remained consistent. Specially, younger and female individuals with lower BMI had the increased HRs. Furthermore, CTI was found to be notably associated with the trajectories of multiple diseases related to CMM, including transitions from a disease-free state to FCMD, from a disease-free state to death and from FCMD to CMM. Specifically, each one-unit increase in the CTI was associated with a 56%, 11%, and 22% higher risk in these transition phases, respectively. In disease-specific analyses, the CTI showed strong associations with transitions from the disease-free state to T2D and CHD. Additionally, patients with stroke and CHD displayed a higher risk of progression from FCMD to CMM. Sensitivity analysis provided more evidence to support the robustness of our findings.

Our data are consistent with those of previous studies on the relationship of the CTI with T2D, CHD, and stroke [24, 26]. However, most of these previous studies primarily focused on a single disease occurrence and not on the role of the CTI in dynamic progression of CMM, including a disease-free state and sequential transitions from CMD onset to CMM and death. In this study, we explored the predictive capacity of CTI in CMM and its component diseases and further investigate if CTI can provide incremental predictive value over CRP and TyG. ROC curve analysis demonstrated that CTI has the strongest predictive value for T2D risk, followed by CMM and FCMD. In C-index, NRI and IDI analyses, CTI showed the incremental predictive value beyond the TyG index and CRP for CHD, FCMD and CMM incident. Although the improvements in these examinations were slight, but they remained statistically significant, implying the advantages of CTI in risk stratification.

On the other hand, several studies have investigated the relationship of the IR (TyG index) and its derived indices with CMM related diseases [12], however, further validations and expansions are required to enhance the prognostic value of biomarker reflecting single physiological state under complex metabolic conditions. For instance, the association between TyG and cardiovascular risk can be influenced by diabetes and hyperlipidemic state [38]. Different from most reports before, we used a multi-state model to explore the role of CTI [26, 35] in disease stage of CMM progression. We found that the CTI had the strongest influence on the transition from a disease-free state to FCMD and played a significant role in the transition from FCMD to CMM. In disease-specific analyses, regardless of whether the CTI was treated as a categorical or continuous variable, a high CTI showed the most pronounced effect on the transition from baseline to T2D and strongly influenced the transition from baseline to CHD. Additionally, significant effects on the pathways from stroke to CMM and from CHD to CMM were observed, indicating the increased susceptibility among these patients. Moreover, C-index value of CTI displayed consistently acceptable across different disease-transition states, suggesting its robust predictive performance in multi-state modeling. Notably, CTI showed strength in predicting CMM progression among patients with FCMD, highlighting its potential as an important tool for risk stratification in this population. These findings provide novel evidence supporting the role of the CTI in CMM progression and emphasize its potential value for early screening and prevention of CMM.

As a combined index reflecting both IR and inflammation, the CTI is linked to CVD risk through several mechanisms. For instance, IR can inhibit the phosphatidylinositol 3-kinase/protein kinase B signaling pathway, decreasing the activity of endothelial nitric oxide synthase and nitric oxide production, which impairs vascular dilation and leads to endothelial dysfunction [39]. Additionally, CRP can increase the expression of adhesion molecules (such as vascular adhesion molecule 1 and intercellular adhesion molecule 1) on endothelial cells, promoting leukocyte adhesion and migration, which accelerates the formation of atherosclerotic plaques [40]. CRP can also bind to oxidized low-density lipoprotein and damaged cell membranes, triggering the classical complement pathway, which enhances local inflammation and supports plaque progression and instability [41]. However, the TyG index and CRP alone reflect a single pathological dimension (glucose–lipid dysregulation or inflammation, respectively) whereas the dual-biomarker framework of the CTI enables a more comprehensive and synchronised assessment of cross-system interactions. Given the substantial inter-individual variability in biomarker expression, CTI, as a integration of TyG index and CRP, offers complementary pathophysiological insights by capturing metabolic dysfunction and systemic inflammation [42, 43]. Therefore, it improves the precision of identifying the population at high risk.

of adverse cardiovascular outcomes.

From a clinical perspective, the CTI could be calculated from existing blood tests including fasting glucose, triglycerides, and CRP and does not require additional examinations or images, thereby having the advantages of applicability in standard healthcare settings. Our study further explores the potential clinical application of CTI. First, in population screening, the CTI can help identify individuals with an increased risk of CMM and its component diseases. RCS analyses demonstrated a clear dose-response relationship between the CTI and cardiometabolic outcomes. In stratified analysis, we further confirmed the consistent association of CTI with T2D, CHD, FCMD, CMM and death across age, gender and BMI. Particularly, younger (< 60 years old), female and lower BMI (< 30 kg/m^2^) individuals had an enhanced HRs compared to elder (≥ 60 years old) male and higher BMI (≥ 30 kg/m^2^) individuals. These findings underscore the clinical importance for closer monitoring and early preventive strategies for those populations once CTI level exceeds ROC-based optimal cut-offs. However, these optimal cut-off values should be considered as cohort-specific points rather than universal cut-off values.

Second, the CTI may provide additional value for risk stratification in patients following the occurrence of FCMD. Our results from multi-state models showed that patients with medium and highest CTI exhibited a significantly higher likelihood of progression to CMM and death requiring intensified monitoring and tailored administration. Although CTI showed the incremental predictive value over TyG index and CRP, the magnitude of improvement was limited. Therefore, CTI could be incorporated into the existing cardiovascular disease risk score to further reflect the metabolic–inflammatory burden. For FCMD patients with increased CTI, stricter management to control lipid and glucose, together with anti-inflammatory treatment such as colchicine might be an effective strategy to improve long-term prognosis.

This study has some limitations. First, the UKB data may not fully represent the broader UK population owing to its participant response rate of only 5.5% [44], and as it is based on a voluntary survey rather than a rigorously controlled sampling process, its generalizability is limited. Second, although we utilized data from a large-scale UKB prospective cohort study, some constraints in data collection hindered our ability to draw definitive causal conclusions. Third, we did not account for lipid level, blood pressure, glycemic status, or medication data, which could have introduced potential bias; future research should address this limitation. Fourth, the CTI measurement may have been influenced by participants’ health status at the time of data collection, potentially introducing reverse causality, which requires further verification. Fifth, the included covariates were selected basing on previous cardiovascular inference studies using UKB. We considered some covariates such as diet, physical activity, sedentary behavior, and sleep quality to be potential mediators rather than confounders, as these factors may lie on the causal pathway between IR/inflammation and cardiometabolic outcomes. Thus, there is a potential for overadjustment bias, attenuating the strength of association for CTI and all outcomes. However, we built separated multivariable models to assess the robustness of our findings. The similar results from minimally adjusted (crude model) and full adjusted model were observed. Sixth, due to the limited CTI measurements (only for baseline) in UK Biobank dataset, it was difficult to capture trajectories over time and trace the temporal association between CTI changes and CMM progression. Further evaluations with CTI measurements at different time points during follow-up are required to validate and extend our findings. Finally, the progression of CMM is affected by various factors, and despite controlling for major covariates, residual confounding due to measurement errors or unmeasured factors may have impacted the results.

## Conclusion

Using data from a large-scale prospective cohort, we reported a positive correlation between the CTI and development of CMM-related diseases. It provided statistically incremental predictive value beyond its individual components TyG and CRP, showing a combined strategy targeting.

cardiometabolic-inflammatory burden to identify individuals at high risk of incidence and progression of CMM. Moreover, CTI was associated with increased risks of transitioning from a disease-free state to FCMD, CMM, and death. These findings suggested that CTI could serve as a simple and scalable tool for risk stratification in CMM-related diseases, highlighting the importance of CTI for early screening. Further validations around predictive capacity of CTI across diverse cohorts and clinical settings are required.

## Supplementary Information

Below is the link to the electronic supplementary material.

Supplementary Material 1

## Data Availability

Data may be obtained from a third party and are not publicly available. The UK Biobank data are available on application at https://www.ukbiobank.ac.uk.

## Abbreviations

BMI: Body mass index
CHD: Coronary heart disease
CI: Confidence interval
CMD: Cardiometabolic disease
CMM: Cardiometabolic multimorbidity
CRP: C-reactive protein
CTI: C-reactive protein-triglyceride-glucose index
CVD: Cardiovascular disease
FCMD: First cardiometabolic disease
FPG: Fasting plasma glucose
HR: Hazard ratio
ICD: International classification of diseases
IR: Insulin resistance
SD: Standard deviation
T2D: Type 2 diabetes
TG: Triglyceride
TyG: Triglyceride-glucose
UKB: UK Biobank
