# Supplementary material for: Associations between the C-reactive protein-triglyceride glucose index and the incidence and progression trajectory of cardiometabolic multimorbidity: a multi-state model study
Source: Cardiovasc Diabetol. 2026 Apr 5;25:125. doi: 10.1186/s12933-026-03174-4 (PMC13085370; doi:10.1186/s12933-026-03174-4)
Supplement: Supplementary file 1 — Supplementary Material 1 [file 12933_2026_3174_MOESM1_ESM.docx]

**Supplementary Material**

**Content**

**Supplementary Table S1.** Detailed information on healthy lifestyle behaviors in UK Biobank

**Supplementary Table S2.** Details of diet definition

**Supplementary Table S3.** Detailed information T2D, stroke, CHD and others’ covariates in UK Biobank

**Supplementary Table S4.** IDI and NRI Values in Different Diseases Among Various Indices (CTI, TyG, and CRP)

**Supplementary Table S5.** Age-Stratified Analysis of the Association Between CTI and T2D, Stroke, CHD, FCMD, CMM, and Mortality (Total *N*=266,049)

**Supplementary Table S6.** Sex-Stratified Analysis of the Association Between CTI and T2D, Stroke, CHD, FCMD, CMM, and Mortality (Total *N*=266,049)

**Supplementary Table S7.** BMI-Stratified Analysis of the Association Between CTI and T2D, Stroke, CHD, FCMD, CMM, and Mortality (Total *N*=266,049)

**Supplementary Table S8.** C-index Values for Transitions from Baseline to T2D, Stroke, CHD, CMM, and Death in the Multi-State Model

**Supplementary Table S9.** C-index Values for Transitions from Baseline to FCMD, CMM, and Death in the Multi-State Model

**Supplementary Table S10**. Association between CTI and T2D, Stroke, CHD, FCMD, CMM, and Death, excluding participants diagnosed FCMD within 2 years of follow-up (Total *N*=262,031)

**Supplementary Table S11**. Associations of CTI with Transitions from Baseline to FCMD, CMM, and Death, and Death, excluding participants diagnosed FCMD within 2 years of follow-up

**Supplementary Table S12**. Associations of CTI with Transitions from Baseline to T2D, stroke, CHD, CMM, and Death, and Death, excluding participants diagnosed FCMD within 2 years of follow-up

**Supplementary Table S13.** Association between CTI and T2D, Stroke, CHD, FCMD, and CMM, considering death as a competing risk event (Fine–Gray Competing Risk Model)

**Supplementary Table S14**. Association between CTI and T2D, Stroke, CHD, FCMD, CMM, and Death, with 5 Imputations for Missing Data (Total *N*=388,926)

**Supplementary Figure S1**. Kaplan–Meier curves for incident CTI and incident T2D, stroke CHD, CMM and Death according to CTI

**Supplementary Figure S2**. ROC Curve of the CTI Values for T2D, stroke, CHD, FCMD, CMM and Death.

**Supplementary Table S1. Detailed information on 5 healthy lifestyle behaviors in UK Biobank**

| **Healthy lifestyle behavior** | **Source and definition** | **UK Biobank field code** |
| --- | --- | --- |
| Alcohol consumption | UK Biobank questionnaire at baseline; alcohol consumption was categorized as never, previous, or current alcohol consumption. | 20117 |
| Smoke status | UK Biobank questionnaire at baseline; smoking status was categorized as never, previous, or current smoking. | 20116 |
| Adequate sleep duration | UK Biobank questionnaire at baseline; ACE touchscreen question "About how many hours sleep do you get in every 24 hours? (please include naps)". Participants who had 7-8 hours’ sleep were defined as ‘Adequate sleep duration’. | 1160 |
| Regular physical activity | UK Biobank questionnaire at baseline; participants who had regular physical activity were defined as those who had more than 150 minutes moderate activity per week, or more than 75 minutes vigorous activity per week, or moderate physical activity at least 5 days a week, or vigorous activity once a week. | 894, 914, 884, 904 |
| Healthy sedentary activity | UK Biobank questionnaire at baseline; participants who had healthy sedentary activity were defined as those who spent less than or equal to 6 hours per day on sedentary activities (driving, watching TV, computer use). | 1090, 1080, 1070 |

**Supplementary Table S2. Details of diet definition^1-3^**

| **Diet component** | **Intake goal** | **Field ID in UK Biobank** | **Amount per serving** |
| --- | --- | --- | --- |
| Fruits | ≥3 servings/day | 1309 (pieces fresh fruit/day)  1319 (pieces dried fruit/day) | 1309 – 1 piece  1319 – 5 pieces |
| Vegetable | ≥3 servings/day | 1289 (tablespoons cooked vegetables/day)  1299 (salad/raw vegetables/day) | 3 heaped tablespoons |
| Fish | ≥2 servings/week | 1329 (oily fish/week)  1339 (non-oily fish/week) | once/week |
| Whole grains | ≥3 servings/day | 1438, 1448 (wholemeal or wholegrain bread slices/week)  1458, 1468 (bran/oat/muesli cereal bowls/week) | 1438/1448 – 1 slice/day  1458/1468 – 1 bowl/day |
| Refined grains | ≤2 servings/day | 1438, 1448 (white, brown, other bread slices/week)  1458, 1468 (biscuit, other cereals/week) | 1438/1448 – 1 slice/day  1458/1468 – 1 bowl/day |
| Processed meats | ≤1 servings/week | 1349 (processed meat/week or daily) | 1349 - once/week |
| Unprocessed red meats | ≤2 servings/week | 1369 (beef/week or day)  1379 (lamb or mutton/week or day)  1389 (pork/week or day) | 1359 - 1389 – once/week |

A healthy diet pattern was defined as meeting four to seven of the above goals.

**Supplementary Table S3. Detailed information T2D, stroke, CHD and others’ covariates in UK Biobank**

| **Factor** | **Source and definition** | **UK Biobank field code** |
| --- | --- | --- |
| T2D | ICD-10 Code: E11 | 130708 |
| Stroke | ICD-10 codes: I60-I64, I69 | 131360, 131362, 131364, 131366, 131368, 131378 |
| CHD | ICD-10 codes: I20-I25 | 131296, 131298, 131300, 131302, 131304, 131306 |
| Age | UK Biobank questionnaire at baseline; participants’ age at recruitment. | 21022 |
| Sex | UK Biobank questionnaire at baseline; participants’ gender was categorized as male, or female. | 31 |
| Ethnicity | UK Biobank questionnaire at baseline; participants’ ethnic background was categorized as white, mixed, asian, black, Chinese, or other. | 21000 |
| Place of residence | UK Biobank questionnaire at baseline; participants’ residence status was categorized as urban, or rural. | 20118 |
| Employment status | UK Biobank questionnaire at baseline; participants who were in paid employment or self-employed or doing unpaid or voluntary work were defined as ‘Employed’; participants who were retired were defined as ‘Retired’; participants who were looking after home and/or family, unable to work because of sickness or disability, unemployed, or full or part-time student were defined as ‘Unemployed’. | 6142, 20119 |
| Education level | UK Biobank questionnaire at baseline; participants who had college or university degree, NVQ or HND or HNC or equivalent, or other professional qualifications were defined as ‘College or above’; participants who obtained A levels/AS levels or equivalent, were defined as ‘High school or equivalent’; Participants with O levels/GCSEs or equivalent, CSEs or equivalent, or none were defined as ‘Less than high school’. | 6138 |
| Household income | UK Biobank questionnaire at baseline; participants’ average total household income before tax was categorized as <18000£, 18000-30999£, 31000-51999£, 52000-100000£, and >100000£. | 738 |
| BMI | UK Biobank questionnaire at baseline; BMI value is constructed from height and weight measured during the initial Assessment Centre visit. Participants’ BMI were categorized as <18.5 kg/m^2^, 18.5-24.9 kg/m^2^, 25.0-29.9 kg/m^2^, and ≥30.0 kg/m^2^. | 21001 |
| Townsend deprivation index | Townsend deprivation index calculated immediately prior to participant joining UK Biobank. Based on the preceding national census output areas. Each participant is assigned a score corresponding to the output area in which their postcode is located. | 22189 |

**Supplementary Table S4. IDI and NRI Values in Different Diseases Among Various Indices (CTI, TyG, and CRP)**

| Outcomes | CTI | | TyG |  | CRP |  |
| --- | --- | --- | --- | --- | --- | --- |
|  | 95% *CI* | *P* | 95% *CI* | *P* | 95% *CI* | *P* |
| T2D |  |  |  |  |  |  |
| IDI | 0.018 (0.016,0.020) | <0.001 | 0.021 (0.019,0.023) | <0.001 | 0.001 (<0.001,0.001) | <0.001 |
| NRI | 0.271 (0.256,0.285) | <0.001 | 0.273 (0.258,0.286) | <0.001 | 0.066 (0.049,0.086) | <0.001 |
| Stroke |  |  |  |  |  |  |
| IDI | <0.001 (<0.001,<0.001) | 0.036 | <0.001 (<0.001,<0.001) | 1.333 | <0.001 (<0.001,<0.001) | <0.001 |
| NRI | 0.043 (0.017,0.066) | 0.008 | <0.001 (-0.023,0.030) | 1.250 | 0.064 (0.038,0.092) | <0.001 |
| CHD |  |  |  |  |  |  |
| IDI | 0.001 (<0.001,0.001) | <0.001 | <0.001 (<0.001,<0.001) | <0.001 | <0.001 (<0.001,<0.001) | <0.001 |
| NRI | 0.093 (0.081,0.105) | <0.001 | 0.072 (0.058,0.084) | <0.001 | 0.056 (0.044,0.067) | <0.001 |
| FCMD |  |  |  |  |  |  |
| IDI | 0.007 (0.007,0.008) | <0.001 | 0.006 (0.006,0.007) | <0.001 | 0.001 (0.001,0.001) | <0.001 |
| NRI | 0.158 (0.149,0.168) | <0.001 | 0.143 (0.132,0.153) | <0.001 | 0.076 (0.066,0.085) | <0.001 |
| CMM |  |  |  |  |  |  |
| IDI | 0.001 (0.001,0.001) | <0.001 | 0.001 (<0.001,0.001) | <0.001 | <0.001 (<0.001,<0.001) | 0.044 |
| NRI | 0.216 (0.167,0.262) | <0.001 | 0.200 (0.144,0.246) | <0.001 | 0.091 (0.052,0.144) | <0.001 |
| Death |  |  |  |  |  |  |
| IDI | <0.001 (<0.001,<0.001) | <0.001 | <0.001 (0,<0.001) | 0.970 | 0.002 (0.001,0.002) | <0.001 |
| NRI | 0.089 (0.070,0.106) | <0.001 | 0.002 (-0.016,0.020) | 0.762 | 0.114 (0.098,0.130) | <0.001 |

Note: CTI, TyG, and CRP as continuous variables.

**Supplementary Table S5**. Age-Stratified Analysis of the Association Between CTI and T2D, Stroke, CHD, FCMD, CMM, and Mortality (Total *N*=266,049)

| Category | Age<60 | | | | Age≥60 | | | |
| --- | --- | --- | --- | --- | --- | --- | --- | --- |
|  | Crude model | | Adjusted model | | Crude model | | Adjusted model | |
|  | HR (95%CI) | *P* Value | HR (95%CI) | *P* Value | HR (95%CI) | *P* Value | HR (95%CI) | *P* Value |
| CTI as three groups (Ref.: Low) | | | | |  |  |  |  |
| T2D |  |  |  |  |  |  |  |  |
| Medium | 3.40 (3.09,3.73) | <0.001 | 2.06 (1.86,2.27) | <0.001 | 2.00 (1.84,2.17) | <0.001 | 1.44 (1.33,1.57) | <0.001 |
| High | 11.83 (10.86,12.89) | <0.001 | 4.90 (4.46,5.38) | <0.001 | 5.11 (4.73,5.51) | <0.001 | 2.80 (2.58,3.04) | <0.001 |
| Stroke |  |  |  |  |  |  |  |  |
| Medium | 1.43 (1.29,1.59) | <0.001 | 1.23 (1.10,1.37) | <0.001 | 1.07 (0.99,1.16) | 0.081 | 1.00 (0.92,1.08) | 0.979 |
| High | 1.74 (1.57,1.94) | <0.001 | 1.32 (1.17,1.49) | <0.001 | 1.21 (1.12,1.31) | <0.001 | 1.06 (0.97,1.15) | 0.195 |
| CHD |  |  |  |  |  |  |  |  |
| Medium | 1.91 (1.80,2.03) | <0.001 | 1.48 (1.39,1.57) | <0.001 | 1.30 (1.24,1.37) | <0.001 | 1.15 (1.09,1.21) | <0.001 |
| High | 3.00 (2.83,3.17) | <0.001 | 1.92 (1.80,2.05) | <0.001 | 1.67 (1.60,1.76) | <0.001 | 1.33 (1.26,1.40) | <0.001 |
| FCMD |  |  |  |  |  |  |  |  |
| Medium | 2.08 (1.98,2.18) | <0.001 | 1.52 (1.45,1.60) | <0.001 | 1.35 (1.30,1.41) | <0.001 | 1.14 (1.10,1.19) | <0.001 |
| High | 4.37 (4.19,4.56) | <0.001 | 2.48 (2.37,2.61) | <0.001 | 2.14 (2.06,2.22) | <0.001 | 1.55 (1.49,1.62) | <0.001 |
| CMM |  |  |  |  |  |  |  |  |
| Medium | 3.00 (2.44,3.69) | <0.001 | 1.78 (1.44,2.20) | <0.001 | 1.77 (1.54,2.03) | <0.001 | 1.33 (1.15,1.53) | <0.001 |
| High | 9.07 (7.53,10.92) | <0.001 | 3.55 (2.89,4.34) | <0.001 | 3.68 (3.24,4.17) | <0.001 | 2.14 (1.87,2.45) | <0.001 |
| Death |  |  |  |  |  |  |  |  |
| Medium | 1.41 (1.33,1.50) | <0.001 | 1.26 (1.18,1.34) | <0.001 | 1.16 (1.11,1.21) | <0.001 | 1.06 (1.02,1.11) | 0.007 |
| High | 2.06 (1.94,2.18) | <0.001 | 1.58 (1.48,1.69) | <0.001 | 1.45 (1.39,1.51) | <0.001 | 1.19 (1.14,1.25) | <0.001 |
| CTI as continues | | | | |  |  |  |  |
| T2D | 3.74 (3.63,3.85) | <0.001 | 2.70 (2.61,2.79) | <0.001 | 2.82 (2.74,2.91) | <0.001 | 2.16 (2.08,2.23) | <0.001 |
| Stroke | 1.38 (1.31,1.45) | <0.001 | 1.20 (1.13,1.28) | <0.001 | 1.13 (1.08,1.17) | <0.001 | 1.04 (0.99,1.09) | 0.088 |
| CHD | 1.75 (1.70,1.80) | <0.001 | 1.40 (1.36,1.45) | <0.001 | 1.34 (1.30,1.37) | <0.001 | 1.18 (1.15,1.21) | <0.001 |
| FCMD | 2.31 (2.27,2.36) | <0.001 | 1.80 (1.76,1.84) | <0.001 | 1.65 (1.61,1.68) | <0.001 | 1.40 (1.37,1.43) | <0.001 |
| CMM | 3.23 (3.03,3.45) | <0.001 | 2.20 (2.04,2.38) | <0.001 | 2.25 (2.13,2.38) | <0.001 | 1.73 (1.63,1.84) | <0.001 |
| Death | 1.50 (1.46,1.54) | <0.001 | 1.31 (1.26,1.35) | <0.001 | 1.27 (1.24,1.29) | <0.001 | 1.13 (1.11,1.16) | <0.001 |

Note: T2D, stroke, and CHD refer to the disease incidence over the entire follow-up period, rather than the first occurrence of disease assessed in the FCMD, which differs from the calculation method in Figure 2. The crude model refers to the unadjusted model, while the adjusted model was controlled for sex, ethnicity, BMI, place of residence, employment status, education level, household income, Townsend deprivation index, alcohol consumption, smoking status, sleep quality, regular physical activity, adherence to a healthy diet, and healthy sedentary behavior.

**Supplementary Table S6**. Sex-Stratified Analysis of the Association Between CTI and T2D, Stroke, CHD, FCMD, CMM, and Mortality (Total *N*=266,049)

| Category | Male | | | | Female | | | |
| --- | --- | --- | --- | --- | --- | --- | --- | --- |
|  | Crude model | | Adjusted model | | Crude model | | Adjusted model | |
|  | HR (95%CI) | *P* Value | HR (95%CI) | *P* Value | HR (95%CI) | *P* Value | HR (95%CI) | *P* Value |
| CTI as three groups (Ref.: Low) | | | | |  |  |  |  |
| T2D |  |  |  |  |  |  |  |  |
| Medium | 2.12 (1.95,2.30) | <0.001 | 1.45 (1.33,1.57) | <0.001 | 3.46 (3.13,3.82) | <0.001 | 2.08 (1.88,2.31) | <0.001 |
| High | 5.41 (5.03,5.83) | <0.001 | 2.80 (2.58,3.02) | <0.001 | 12.24 (11.19,13.39) | <0.001 | 5.13 (4.65,5.66) | <0.001 |
| Stroke |  |  |  |  |  |  |  |  |
| Medium | 1.16 (1.06,1.26) | 0.001 | 1.00 (0.91,1.09) | 0.937 | 1.51 (1.38,1.66) | <0.001 | 1.09 (0.98,1.20) | 0.098 |
| High | 1.32 (1.21,1.43) | <0.001 | 1.07 (0.98,1.17) | 0.154 | 1.82 (1.66,2.00) | <0.001 | 1.14 (1.02,1.27) | 0.016 |
| CHD |  |  |  |  |  |  |  |  |
| Medium | 1.45 (1.38,1.52) | <0.001 | 1.22 (1.16,1.28) | <0.001 | 1.78 (1.68,1.90) | <0.001 | 1.26 (1.18,1.35) | <0.001 |
| High | 1.84 (1.75,1.93) | <0.001 | 1.42 (1.35,1.49) | <0.001 | 2.83 (2.66,3.00) | <0.001 | 1.66 (1.55,1.78) | <0.001 |
| FCMD |  |  |  |  |  |  |  |  |
| Medium | 1.50 (1.45,1.56) | <0.001 | 1.20 (1.16,1.25) | <0.001 | 1.98 (1.89,2.08) | <0.001 | 1.35 (1.28,1.42) | <0.001 |
| High | 2.40 (2.31,2.49) | <0.001 | 1.66 (1.60,1.73) | <0.001 | 4.10 (3.93,4.28) | <0.001 | 2.17 (2.06,2.28) | <0.001 |
| CMM |  |  |  |  |  |  |  |  |
| Medium | 2.02 (1.76,2.32) | <0.001 | 1.37 (1.19,1.58) | <0.001 | 2.62 (2.14,3.20) | <0.001 | 1.52 (1.24,1.87) | <0.001 |
| High | 3.95 (3.47,4.49) | <0.001 | 2.06 (1.80,2.37) | <0.001 | 8.71 (7.28,10.41) | <0.001 | 3.58 (2.94,4.36) | <0.001 |
| Death |  |  |  |  |  |  |  |  |
| Medium | 1.28 (1.22,1.34) | <0.001 | 1.06 (1.01,1.12) | 0.013 | 1.52 (1.44,1.60) | <0.001 | 1.10 (1.05,1.16) | <0.001 |
| High | 1.63 (1.56,1.71) | <0.001 | 1.23 (1.17,1.29) | <0.001 | 2.10 (2.00,2.21) | <0.001 | 1.29 (1.22,1.36) | <0.001 |
| CTI as continues | | | | |  |  |  |  |
| T2D | 2.84 (2.76,2.92) | <0.001 | 2.19 (2.12,2.26) | <0.001 | 3.86 (3.74,3.98) | <0.001 | 2.80 (2.70,2.90) | <0.001 |
| Stroke | 1.18 (1.13,1.23) | <0.001 | 1.06 (1.01,1.11) | <0.001 | 1.41 (1.35,1.48) | <0.001 | 1.10 (1.04,1.17) | 0.001 |
| CHD | 1.38 (1.35,1.41) | <0.001 | 1.21 (1.18,1.24) | <0.001 | 1.73 (1.68,1.78) | <0.001 | 1.34 (1.29,1.38) | <0.001 |
| FCMD | 1.72 (1.69,1.76) | <0.001 | 1.46 (1.43,1.49) | <0.001 | 2.26 (2.21,2.31) | <0.001 | 1.72 (1.67,1.76) | <0.001 |
| CMM | 2.23 (2.12,2.35) | <0.001 | 1.69 (1.60,1.80) | <0.001 | 3.27 (3.06,3.50) | <0.001 | 2.37 (2.18,2.57) | <0.001 |
| Death | 1.33 (1.30,1.36) | <0.001 | 1.14 (1.11,1.17) | <0.001 | 1.53 (1.49,1.57) | <0.001 | 1.20 (1.16,1.23) | <0.001 |

Note: T2D, stroke, and CHD refer to the disease incidence over the entire follow-up period, rather than the first occurrence of disease assessed in the FCMD, which differs from the calculation method in Figure 2. The crude model refers to the unadjusted model, while the adjusted model was controlled for age, ethnicity, BMI, place of residence, employment status, education level, household income, Townsend deprivation index, alcohol consumption, smoking status, sleep quality, regular physical activity, adherence to a healthy diet, and healthy sedentary behavior.

**Supplementary Table S7**. BMI-Stratified Analysis of the Association Between CTI and T2D, Stroke, CHD, FCMD, CMM, and Mortality (Total *N*=266,049)

| Category | BMI<30 | | | | BMI≥30 | | | |
| --- | --- | --- | --- | --- | --- | --- | --- | --- |
|  | Crude model | | Adjusted model | | Crude model | | Adjusted model | |
|  | HR (95%CI) | *P* Value | HR (95%CI) | *P* Value | HR (95%CI) | *P* Value | HR (95%CI) | *P* Value |
| CTI as three groups (Ref.: Low) | | | | |  |  |  |  |
| T2D |  |  |  |  |  |  |  |  |
| Medium | 2.44 (2.26,2.63) | <0.001 | 1.94 (1.80,2.09) | <0.001 | 1.57 (1.39,1.76) | <0.001 | 1.50 (1.33,1.69) | <0.001 |
| High | 6.56 (6.11,7.03) | <0.001 | 4.60 (4.28,4.94) | <0.001 | 3.34 (2.99,3.73) | <0.001 | 3.04 (2.72,3.40) | <0.001 |
| Stroke |  |  |  |  |  |  |  |  |
| Medium | 1.38 (1.29,1.47) | <0.001 | 1.03 (0.96,1.10) | 0.420 | 1.27 (1.05,1.55) | 0.016 | 1.08 (0.89,1.32) | 0.431 |
| High | 1.74 (1.62,1.86) | <0.001 | 1.14 (1.06,1.23) | <0.001 | 1.31 (1.08,1.57) | 0.005 | 1.05 (0.87,1.27) | 0.624 |
| CHD |  |  |  |  |  |  |  |  |
| Medium | 1.72 (1.65,1.80) | <0.001 | 1.30 (1.25,1.36) | <0.001 | 1.28 (1.15,1.43) | <0.001 | 1.10 (0.99,1.23) | 0.078 |
| High | 2.47 (2.36,2.57) | <0.001 | 1.62 (1.56,1.70) | <0.001 | 1.63 (1.47,1.80) | <0.001 | 1.32 (1.19,1.46) | <0.001 |
| FCMD |  |  |  |  |  |  |  |  |
| Medium | 1.74 (1.68,1.80) | <0.001 | 1.33 (1.28,1.37) | <0.001 | 1.40 (1.30,1.51) | <0.001 | 1.27 (1.17,1.37) | <0.001 |
| High | 2.89 (2.80,2.99) | <0.001 | 1.94 (1.87,2.01) | <0.001 | 2.25 (2.10,2.43) | <0.001 | 1.94 (1.80,2.09) | <0.001 |
| CMM |  |  |  |  |  |  |  |  |
| Medium | 2.31 (2.02,2.64) | <0.001 | 1.62 (1.42,1.86) | <0.001 | 1.40 (1.10,1.77) | 0.005 | 1.19 (0.94,1.51) | 0.142 |
| High | 5.30 (4.68,6.00) | <0.001 | 3.11 (2.74,3.54) | <0.001 | 2.51 (2.02,3.13) | <0.001 | 1.97 (1.57,2.45) | <0.001 |
| Death |  |  |  |  |  |  |  |  |
| Medium | 1.45 (1.39,1.50) | <0.001 | 1.04 (1.00,1.07) | 0.074 | 1.32 (1.19,1.47) | <0.001 | 1.08 (0.97,1.20) | 0.155 |
| High | 2.01 (1.94,2.09) | <0.001 | 1.21 (1.16,1.26) | <0.001 | 1.58 (1.43,1.75) | <0.001 | 1.19 (1.08,1.31) | 0.001 |
| CTI as continues | | | | |  |  |  |  |
| T2D | 3.08 (2.99,3.18) | <0.001 | 2.64 (2.56,2.73) | <0.001 | 2.46 (2.38,2.54) | <0.001 | 2.35 (2.27,2.43) | <0.001 |
| Stroke | 1.39 (1.34,1.45) | <0.001 | 1.09 (1.05,1.14) | <0.001 | 1.14 (1.05,1.22) | 0.001 | 1.04 (0.96,1.13) | 0.300 |
| CHD | 1.66 (1.63,1.70) | <0.001 | 1.33 (1.30,1.36) | <0.001 | 1.28 (1.23,1.33) | <0.001 | 1.17 (1.13,1.22) | <0.001 |
| FCMD | 1.88 (1.85,1.91) | <0.001 | 1.53 (1.50,1.55) | <0.001 | 1.80 (1.76,1.85) | <0.001 | 1.70 (1.66,1.75) | <0.001 |
| CMM | 2.68 (2.53,2.84) | <0.001 | 2.11 (1.98,2.24) | <0.001 | 1.96 (1.83,2.10) | <0.001 | 1.78 (1.66,1.92) | <0.001 |
| Death | 1.52 (1.49,1.55) | <0.001 | 1.13 (1.11,1.16) | <0.001 | 1.28 (1.23,1.33) | <0.001 | 1.14 (1.10,1.19) | <0.001 |

Note: T2D, stroke, and CHD refer to the disease incidence over the entire follow-up period, rather than the first occurrence of disease assessed in the FCMD, which differs from the calculation method in Figure 2. The crude model refers to the unadjusted model, while the adjusted model was controlled for age, sex, ethnicity, place of residence, employment status, education level, household income, Townsend deprivation index, alcohol consumption, smoking status, sleep quality, regular physical activity, adherence to a healthy diet, and healthy sedentary behavior.

**Supplementary Table S8.** **C-index Values for Transitions from Baseline to T2D, Stroke, CHD, CMM, and Death in the Multi-State Model**

| Transition path | C-index | 95%CI | |
| --- | --- | --- | --- |
|  |  | Lower | Upper |
| Baseline → T2D | 0.8024 | 0.7990 | 0.8058 |
| Baseline → Stroke | 0.7049 | 0.6980 | 0.7117 |
| Baseline → CHD | 0.7126 | 0.7091 | 0.7160 |
| Baseline → Death | 0.7287 | 0.7247 | 0.7328 |
| T2D → CMM | 0.6246 | 0.6103 | 0.6390 |
| T2D → Death | 0.6560 | 0.6415 | 0.6705 |
| Stroke → CMM | 0.6380 | 0.6126 | 0.6634 |
| Stroke → Death | 0.6228 | 0.6045 | 0.6411 |
| CHD → CMM | 0.6435 | 0.6287 | 0.6582 |
| CHD → Death | 0.6920 | 0.6809 | 0.7032 |
| CMM → Death | 0.6487 | 0.6304 | 0.6671 |

Note: CTI as a continuous variable

**Supplementary Table S9.** **C-index Values for Transitions from Baseline to FCMD, CMM, and Death in the Multi-State Model**

| Transition path | C-index | 95%CI | |
| --- | --- | --- | --- |
|  |  | Lower | Upper |
| Baseline → FCMD | 0.7247 | 0.7223 | 0.7272 |
| Baseline → Death | 0.7287 | 0.7247 | 0.7328 |
| FCMD → CMM | 0.6292 | 0.6196 | 0.6387 |
| FCMD → Death | 0.6644 | 0.6566 | 0.6722 |
| CMM → Death | 0.6487 | 0.6304 | 0.6671 |

Note: CTI as a continuous variable

**Supplementary Table S10**. Association between CTI and T2D, Stroke, CHD, FCMD, CMM, and Death, excluding participants diagnosed FCMD within 2 years of follow-up (Total *N*=262,031)

| Category | Crude model | | Adjusted model | |
| --- | --- | --- | --- | --- |
|  | HR (95%CI) | *P* Value | HR (95%CI) | *P* Value |
| CTI as three groups (Ref.: Low) | | | | |
| T2D |  |  |  |  |
| Medium | 2.83 (2.65,3.03) | <0.001 | 1.70 (1.59,1.83) | <0.001 |
| High | 8.29 (7.80,8.81) | <0.001 | 3.57 (3.35,3.82) | <0.001 |
| Stroke |  |  |  |  |
| Medium | 1.37 (1.28,1.46) | <0.001 | 1.03 (0.96,1.10) | 0.224 |
| High | 1.60 (1.49,1.70) | <0.001 | 1.08 (1.01,1.17) | 0.004 |
| CHD |  |  |  |  |
| Medium | 1.74 (1.67,1.81) | <0.001 | 1.27 (1.22,1.32) | <0.001 |
| High | 2.38 (2.29,2.48) | <0.001 | 1.50 (1.44,1.57) | <0.001 |
| FCMD |  |  |  |  |
| Medium | 1.83 (1.78,1.89) | <0.001 | 1.28 (1.24,1.32) | <0.001 |
| High | 3.23 (3.13,3.32) | <0.001 | 1.84 (1.78,1.90) | <0.001 |
| CMM |  |  |  |  |
| Medium | 2.49 (2.19,2.83) | <0.001 | 1.44 (1.27,1.65) | <0.001 |
| High | 5.78 (5.14,6.49) | <0.001 | 2.44 (2.15,2.76) | <0.001 |
| Death |  |  |  |  |
| Medium | 1.46 (1.41,1.51) | <0.001 | 1.08 (1.04,1.12) | <0.001 |
| High | 1.93 (1.87,2.00) | <0.001 | 1.24 (1.19,1.28) | <0.001 |
| CTI as continues | | | | |
| T2D | 3.31 (3.24,3.38) | <0.001 | 2.42 (2.36,2.48) | <0.001 |
| Stroke | 1.32 (1.28,1.37) | <0.001 | 1.08 (1.04,1.12) | <0.001 |
| CHD | 1.57 (1.55,1.60) | <0.001 | 1.25 (1.22,1.28) | <0.001 |
| FCMD | 1.97 (1.95,2.00) | <0.001 | 1.53 (1.51,1.56) | <0.001 |
| CMM | 2.60 (2.48,2.73) | <0.001 | 1.83 (1.73,1.93) | <0.001 |
| Death | 1.45 (1.43,1.48) | <0.001 | 1.15 (1.13,1.18) | <0.001 |

Note: T2D, stroke, and CHD refer to the disease incidence over the entire follow-up period, rather than the first occurrence of disease assessed in the FCMD, which differs from the calculation method in Figure 2. The crude model refers to the unadjusted model, while the adjusted model was controlled for age, sex, ethnicity, BMI, place of residence, employment status, education level, household income, Townsend deprivation index, alcohol consumption, smoking status, sleep quality, regular physical activity, adherence to a healthy diet, and healthy sedentary behavior.

**Supplementary Table S11**. **Associations of CTI with Transitions from Baseline to FCMD, CMM, and Death, and Death, excluding participants diagnosed FCMD within 2 years of follow-up**

| Groups | Baseline → FCMD | Baseline → Death | FCMD → CMM | FCMD → Death | CMM → Death |
| --- | --- | --- | --- | --- | --- |
| CTI as three groups (Ref.: Low) | | | | | |
| Medium | 1.27 (1.23,1.32) *** | 1.06 (1.02,1.11) ** | 1.14 (1.00,1.30) * | 0.90 (0.83,0.98) * | 0.97 (0.76,1.23) |
| High | 1.83 (1.77,1.89) *** | 1.18 (1.13,1.23) *** | 1.37 (1.20,1.55) *** | 0.90 (0.83,0.98) * | 1.01 (0.80,1.27) |
| CTI as continues | | | | | |
| CTI | 1.53 (1.50,1.55) *** | 1.11 (1.09,1.14) *** | 1.19 (1.13,1.26) *** | 0.97 (0.93,1.01) | 0.98 (0.89,1.08) |

Note: *** *P*<0.001, ***P*<0.01, * *P*<0.05

All models were adjusted for age, sex, ethnicity, BMI, place of residence, employment status, education level, household income, Townsend deprivation index, alcohol consumption, smoking status, sleep quality, regular physical activity, adherence to a healthy diet, and healthy sedentary behavior.

**Supplementary Table S12**. **Associations of CTI with Transitions from Baseline to T2D, stroke, CHD, CMM, and Death, and Death, excluding participants diagnosed FCMD within 2 years of follow-up**

| Transition path | CTI as three groups (Ref.: Low) | | CTI as continues |
| --- | --- | --- | --- |
|  | Medium | High |  |
| Baseline → T2D | 1.72 (1.60,1.85) *** | 3.66 (3.41,3.92) *** | 2.41 (2.35,2.48) *** |
| Baseline → Stroke | 1.01 (0.93,1.08) | 1.03 (0.96,1.12) | 1.02 (0.98,1.07) |
| Baseline → CHD | 1.26 (1.20,1.31) *** | 1.48 (1.42,1.55) *** | 1.24 (1.21,1.27) *** |
| Baseline → Death | 1.06 (1.02,1.11) ** | 1.18 (1.13,1.23) *** | 1.11 (1.09,1.14) *** |
| T2D → CMM | 1.20 (0.95,1.52) | 1.16 (0.93,1.45) | 1.04 (0.96,1.12) |
| T2D → Death | 0.93 (0.76,1.13) | 0.88 (0.74,1.06) | 0.95 (0.89,1.02) |
| Stroke → CMM | 1.08 (0.81,1.44) | 1.35 (1.01,1.79) * | 1.23 (1.05,1.43) * |
| Stroke → Death | 0.86 (0.75,1.00) | 0.88 (0.76,1.03) | 0.95 (0.87,1.04) |
| CHD → CMM | 1.09 (0.90,1.32) | 1.47 (1.23,1.77) *** | 1.34 (1.23,1.47) *** |
| CHD → Death | 0.99 (0.88,1.12) | 0.98 (0.86,1.11) | 0.99 (0.93,1.07) |
| CMM → Death | 0.97 (0.76,1.23) | 1.01 (0.80,1.27) | 0.98 (0.89,1.08) |

Note: *** *P*<0.001, ***P*<0.01, * *P*<0.05

All models were adjusted for age, sex, ethnicity, BMI, place of residence, employment status, education level, household income, Townsend deprivation index, alcohol consumption, smoking status, sleep quality, regular physical activity, adherence to a healthy diet, and healthy sedentary behavior.

**Supplementary Table S13. Association between CTI and T2D, Stroke, CHD, FCMD, and CMM, considering death as a competing risk event (Fine–Gray Competing Risk Model)**

| Category | Crude model | | Adjusted model | |
| --- | --- | --- | --- | --- |
|  | HR (95%CI) | *P* Value | HR (95%CI) | *P* Value |
| CTI as three groups (Ref.: Low) | | | | |
| T2D |  |  |  |  |
| Medium | 2.79 (2.62,2.97) | <0.001 | 1.69 (1.58,1.80) | <0.001 |
| High | 8.22 (7.77,8.71) | <0.001 | 3.57 (3.35,3.80) | <0.001 |
| Stroke |  |  |  |  |
| Medium | 1.37 (1.29,1.46) | <0.001 | 1.03 (0.97,1.11) | 0.320 |
| High | 1.60 (1.51,1.71) | <0.001 | 1.09 (1.01,1.16) | 0.023 |
| CHD |  |  |  |  |
| Medium | 1.72 (1.65,1.79) | <0.001 | 1.24 (1.20,1.29) | <0.001 |
| High | 2.40 (2.32,2.49) | <0.001 | 1.50 (1.43,1.56) | <0.001 |
| FCMD |  |  |  |  |
| Medium | 1.82 (1.77,1.88) | <0.001 | 1.27 (1.23,1.31) | <0.001 |
| High | 3.26 (3.17,3.36) | <0.001 | 1.84 (1.78,1.90) | <0.001 |
| CMM |  |  |  |  |
| Medium | 2.46 (2.20,2.76) | <0.001 | 1.43 (1.27,1.61) | <0.001 |
| High | 5.84 (5.26,6.48) | <0.001 | 2.45 (2.19,2.75) | <0.001 |
| CTI as continues | | | | |
| T2D | 3.27 (3.20,3.35) | <0.001 | 2.40 (2.34,2.47) | <0.001 |
| Stroke | 1.31 (1.27,1.35) | <0.001 | 1.06 (1.02,1.11) | 0.001 |
| CHD | 1.58 (1.55,1.60) | <0.001 | 1.25 (1.22,1.28) | <0.001 |
| FCMD | 1.99 (1.97,2.02) | <0.001 | 1.55 (1.52,1.57) | <0.001 |
| CMM | 2.64 (2.53,2.75) | <0.001 | 1.87 (1.78,1.97) | <0.001 |

Note: T2D, stroke, and CHD refer to the incidence of these diseases over the entire follow-up period, rather than the first occurrence of disease as assessed in the FCMD, which differs from the calculation method used in Figure 2. Since death was treated as a competing risk, no further calculations were conducted for mortality. The crude model represents the unadjusted estimates, whereas the adjusted model controlled for age, sex, ethnicity, BMI, place of residence, employment status, education level, household income, Townsend deprivation index, alcohol consumption, smoking status, sleep quality, regular physical activity, adherence to a healthy diet, and healthy sedentary behavior.

**Supplementary Table S14**. **Association between CTI and T2D, Stroke, CHD, FCMD, CMM, and Death, with 5 Imputations for Missing Data (Total *N*=388,926)**

| Category | Crude model | | Adjusted model | |
| --- | --- | --- | --- | --- |
|  | HR (95%CI) | *P* Value | HR (95%CI) | *P* Value |
| CTI as three groups (Ref.: Low) | | | | |
| T2D |  |  |  |  |
| Medium | 2.75 (2.61,2.89) | <0.001 | 1.69 (1.60,1.78) | <0.001 |
| High | 8.15 (7.79,8.53) | <0.001 | 3.63 (3.45,3.81) | <0.001 |
| Stroke |  |  |  |  |
| Medium | 1.40 (1.33,1.48) | <0.001 | 1.08 (1.02,1.14) | 0.007 |
| High | 1.64 (1.56,1.73) | <0.001 | 1.14 (1.08,1.20) | <0.001 |
| CHD |  |  |  |  |
| Medium | 1.73 (1.68,1.78) | <0.001 | 1.26 (1.22,1.30) | <0.001 |
| High | 2.45 (2.37,2.52) | <0.001 | 1.53 (1.48,1.58) | <0.001 |
| FCMD |  |  |  |  |
| Medium | 1.84 (1.79,1.88) | <0.001 | 1.29 (1.26,1.32) | <0.001 |
| High | 3.32 (3.24,3.39) | <0.001 | 1.90 (1.85,1.95) | <0.001 |
| CMM |  |  |  |  |
| Medium | 2.44 (2.23,2.68) | <0.001 | 1.44 (1.31,1.58) | <0.001 |
| High | 6.00 (5.52,6.53) | <0.001 | 2.57 (2.35,2.82) | <0.001 |
| Death |  |  |  |  |
| Medium | 1.46 (1.42,1.50) | <0.001 | 1.09 (1.06,1.12) | <0.001 |
| High | 1.98 (1.93,2.04) | <0.001 | 1.27 (1.23,1.31) | <0.001 |
| CTI as continues | | | | |
| T2D | 3.26 (3.21,3.32) | <0.001 | 2.41 (2.37,2.46) | <0.001 |
| Stroke | 1.32 (1.28,1.35) | <0.001 | 1.09 (1.06,1.12) | <0.001 |
| CHD | 1.60 (1.58,1.62) | <0.001 | 1.28 (1.26,1.30) | <0.001 |
| FCMD | 2.01 (1.99,2.04) | <0.001 | 1.58 (1.56,1.60) | <0.001 |
| CMM | 2.68 (2.60,2.77) | <0.001 | 1.93 (1.86,2.01) | <0.001 |
| Death | 1.47 (1.45,1.49) | <0.001 | 1.18 (1.16,1.20) | <0.001 |

Note: T2D, stroke, and CHD refer to the disease incidence over the entire follow-up period, rather than the first occurrence of disease assessed in the FCMD, which differs from the calculation method in Figure 2. The crude model refers to the unadjusted model, while the adjusted model was controlled for age, sex, ethnicity, BMI, place of residence, employment status, education level, household income, Townsend deprivation index, alcohol consumption, smoking status, sleep quality, regular physical activity, adherence to a healthy diet, and healthy sedentary behavior.

**Supplementary
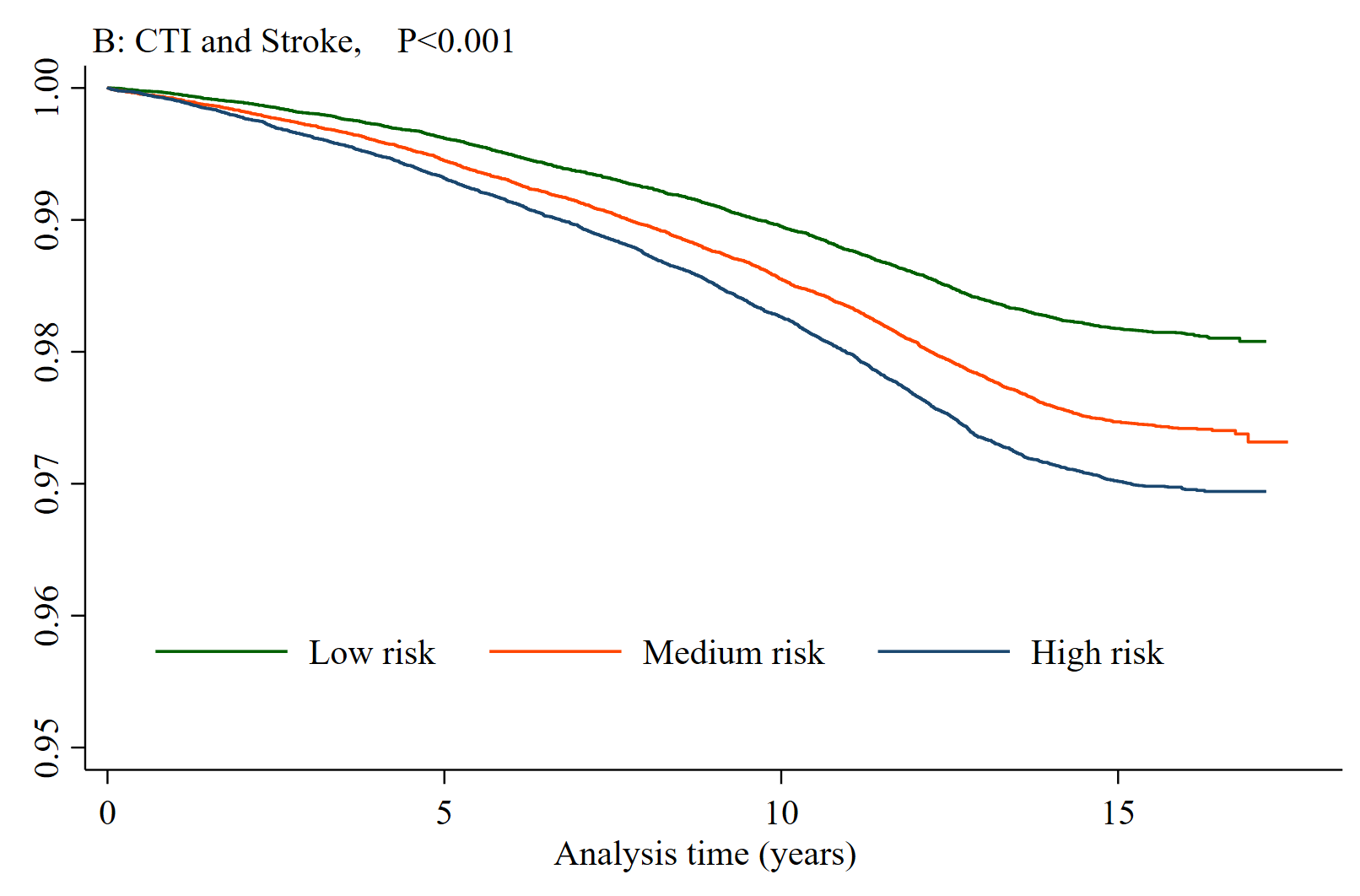

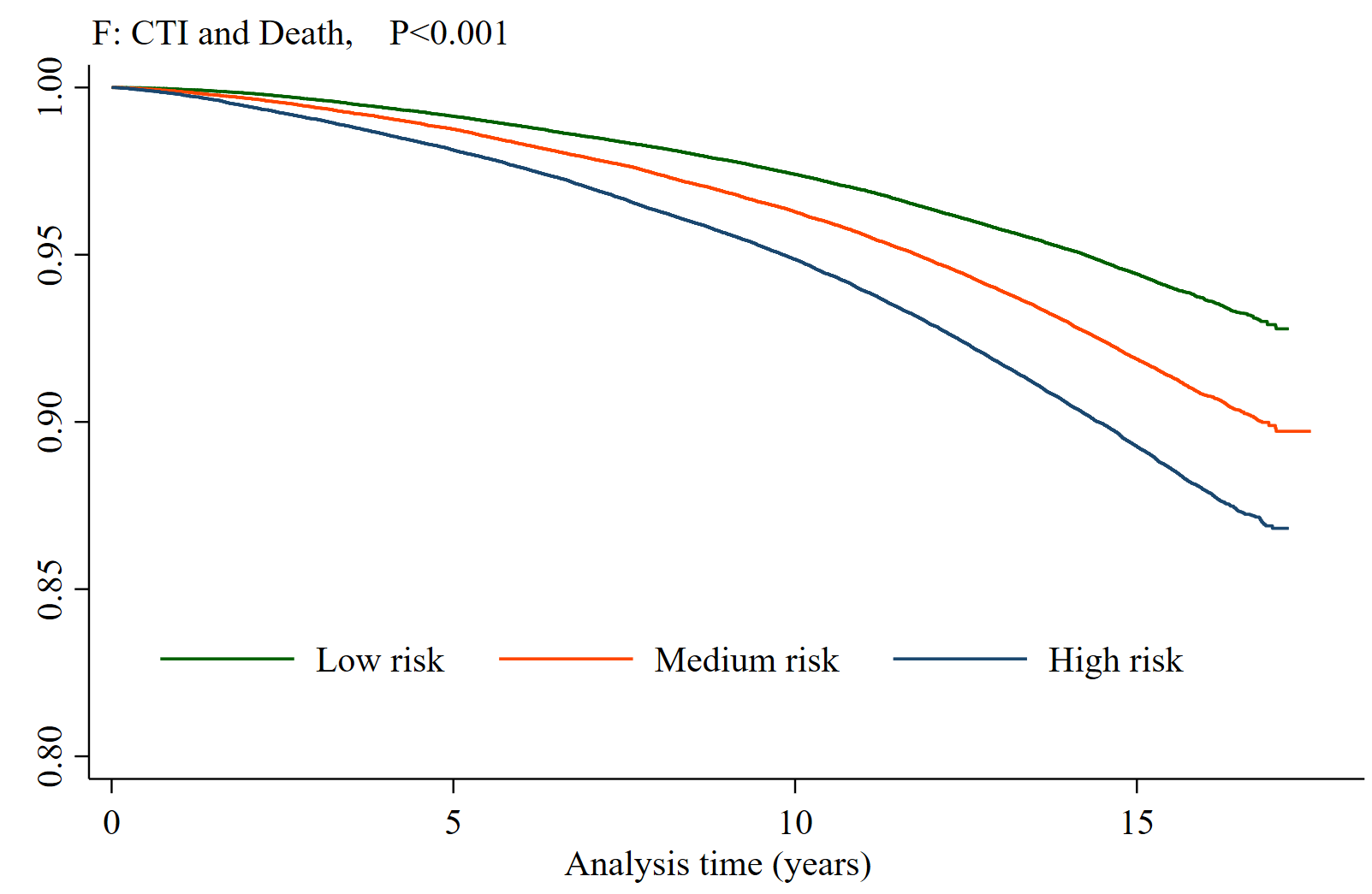

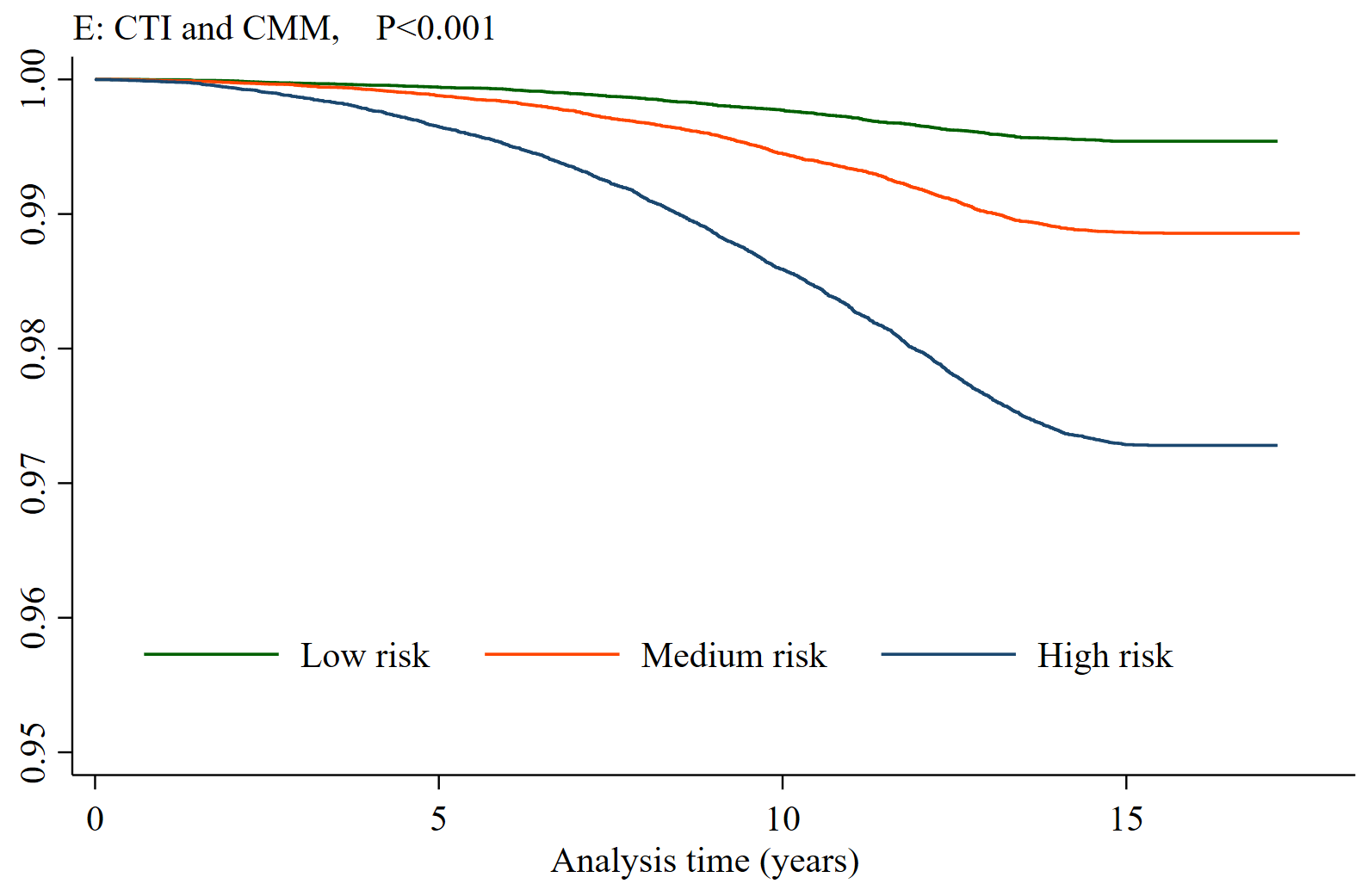

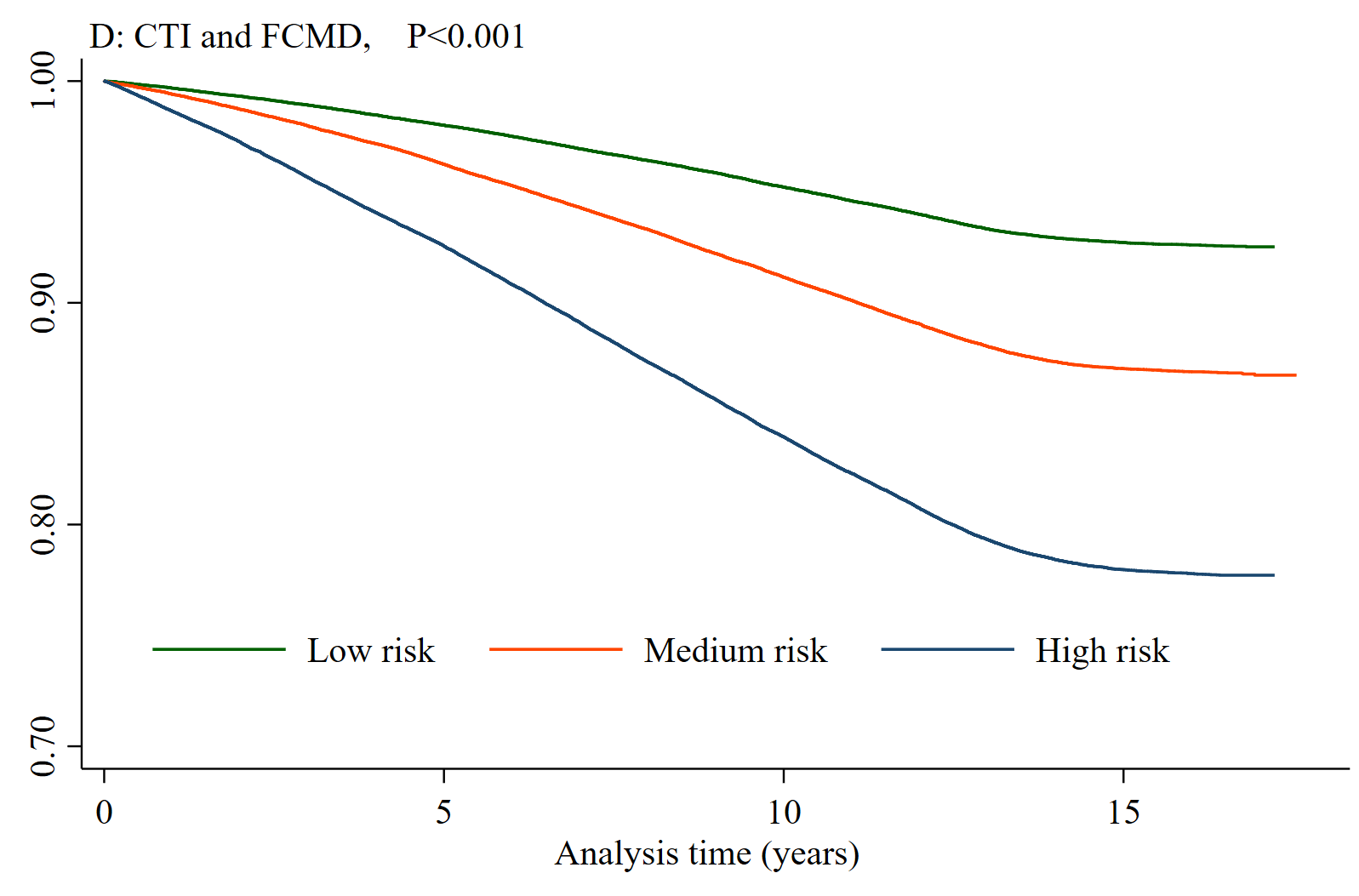

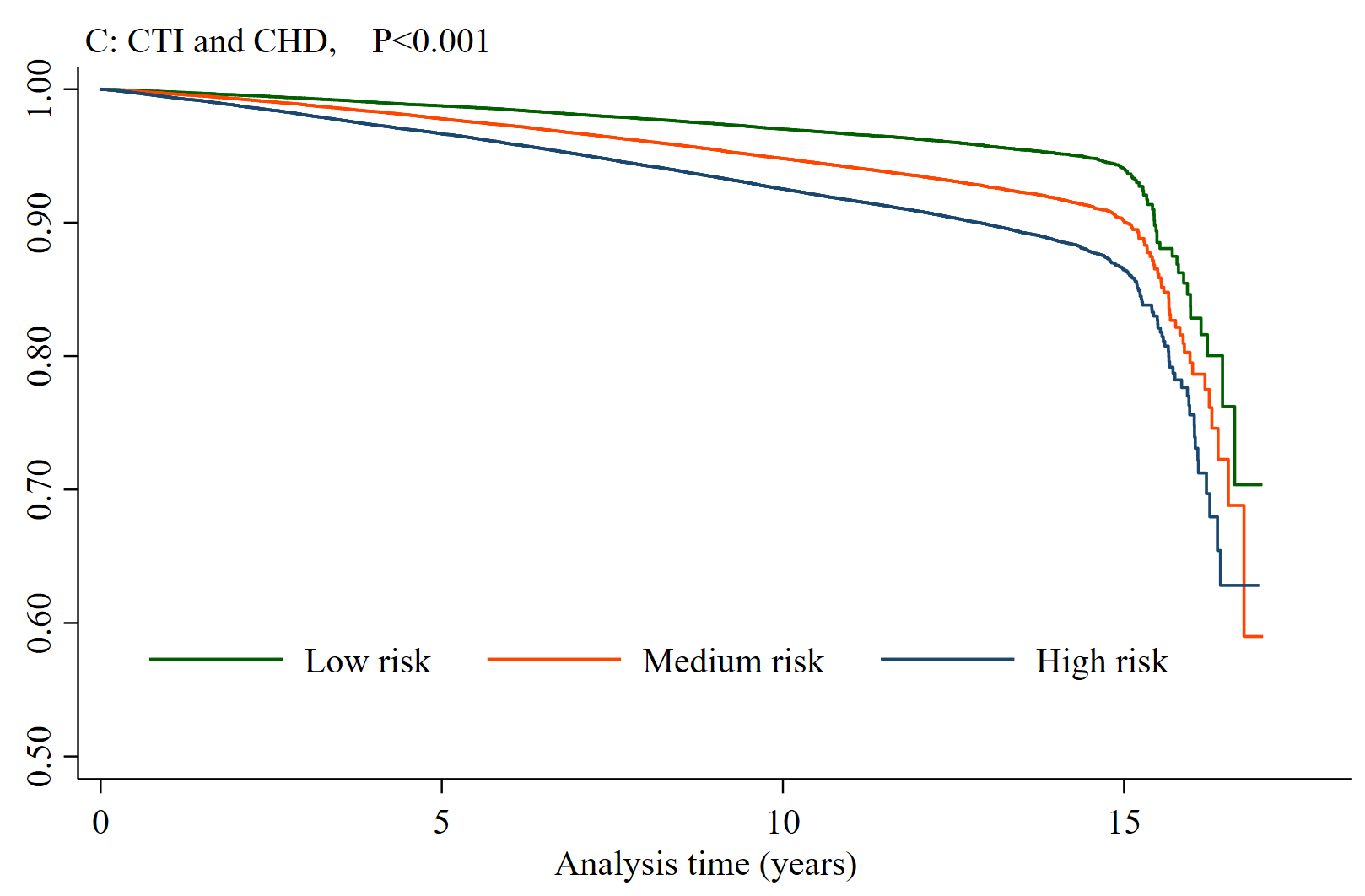

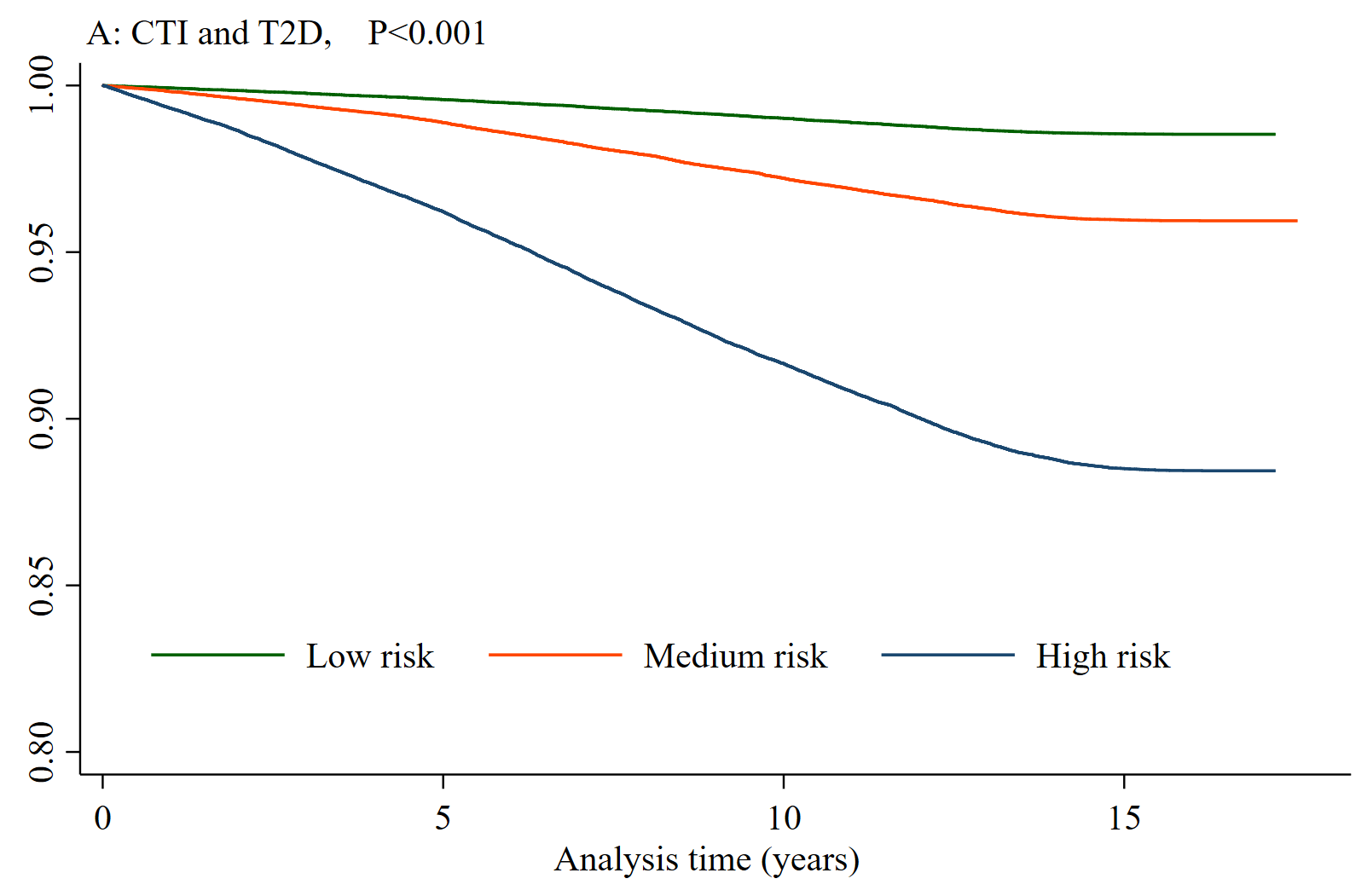
Figure S1. Kaplan–Meier curves for incident CTI and incident T2D, stroke, CHD, FCMD, CMM and Death according to CTI**


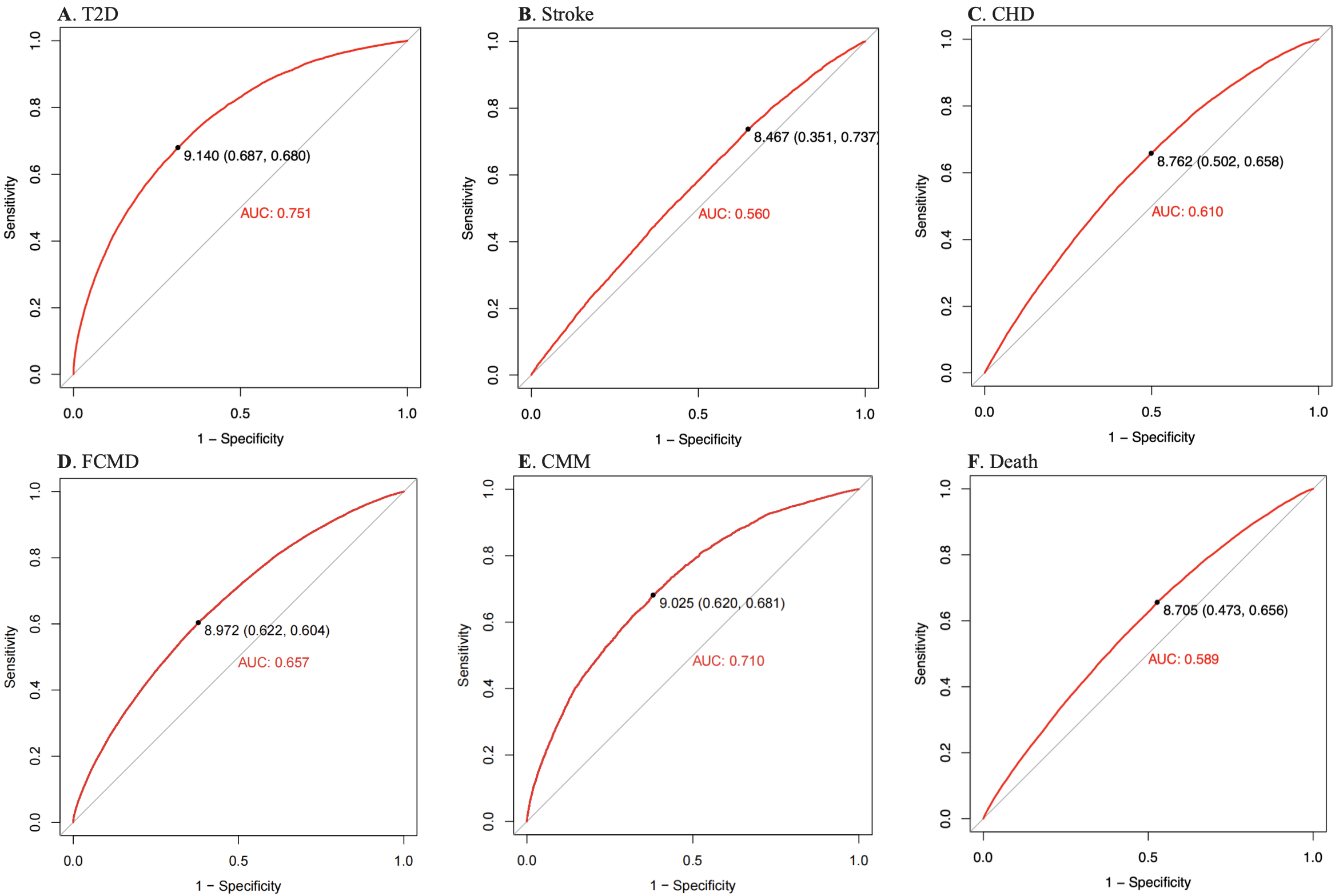


**Supplementary Figure S2**. **ROC Curve of the CTI Values for T2D, stroke, CHD, FCMD, CMM and Death.**
